# Colonization and genetic diversification processes of *Leishmania infantum* in the Americas

**DOI:** 10.1038/s42003-021-01658-5

**Published:** 2021-01-29

**Authors:** Philipp Schwabl, Mariana C. Boité, Giovanni Bussotti, Arne Jacobs, Bjorn Andersson, Otacilio Moreira, Anita L. Freitas-Mesquita, Jose Roberto Meyer-Fernandes, Erich L. Telleria, Yara Traub-Csekö, Slavica Vaselek, Tereza Leštinová, Petr Volf, Fernanda N. Morgado, Renato Porrozzi, Martin Llewellyn, Gerald F. Späth, Elisa Cupolillo

**Affiliations:** 1 School of Life Sciences, Institute of Biodiversity, Animal Health and Comparative Medicine, University of Glasgow, G12 8QQ Glasgow, UK; 2 Laboratório de Pesquisa em Leishmaniose, Instituto Oswaldo Cruz, FIOCRUZ, 21040-365 Rio de Janeiro, Brazil; 3 Institut Pasteur-Bioinformatics and Biostatistics Hub-C3BI, USR 3756 IP CNRS, 75015 Paris, France; 4 Department of Parasites and Insect Vectors, Institut Pasteur, INSERM U1201, Unité de Parasitology moléculaire et Signalisation, 75015 Paris, France; 5 Department of Cell and Molecular Biology, Science for Life Laboratory, Karolinska Institutet, Biomedicum 9C, 171 77 Stockholm, Sweden; 6 Laboratório de Biologia Molecular e Doenças Endêmicas, Instituto Oswaldo Cruz, Fiocruz, 21040-365 Rio de Janeiro, RJ Brazil; 7 Instituto de Bioquímica Médica Leopoldo de Meis (IBqM), Universidade Federal do Rio de Janeiro (UFRJ), 21941-590 Rio de Janeiro, RJ Brazil; 8 Laboratório de Biologia Molecular de Parasitas e Vetores, Instituto Oswaldo Cruz, 21040-365 Rio de Janeiro, Brazil; 9 Faculty of Science, Department of Parasitology, Charles University, 128 44 Prague, Czech Republic

**Keywords:** Parasite genomics, Mutation, Genetic variation, Parasitic infection

## Abstract

*Leishmania infantum* causes visceral leishmaniasis, a deadly vector-borne disease introduced to the Americas during the colonial era. This non-native trypanosomatid parasite has since established widespread transmission cycles using alternative vectors, and human infection has become a significant concern to public health, especially in Brazil. A multi-kilobase deletion was recently detected in Brazilian *L. infantum* genomes and is suggested to reduce susceptibility to the anti-leishmanial drug miltefosine. We show that deletion-carrying strains occur in at least 15 Brazilian states and describe diversity patterns suggesting that these derive from common ancestral mutants rather than from recurrent independent mutation events. We also show that the deleted locus and associated enzymatic activity is restored by hybridization with non-deletion type strains. Genetic exchange appears common in areas of secondary contact but also among closely related parasites. We examine demographic and ecological scenarios underlying this complex *L. infantum* population structure and discuss implications for disease control.

## Abstract (web summary)

Philipp Schwabl, Mariana Boité, and colleagues analyze 126 *Leishmania infantum* genomes to determine how demographic and selective consequences of the parasite’s invasive history have contributed to intricate population genetic heterogeneity across Brazil. Their data suggest a complex interplay of population expansion, secondary contact and genetic exchange events underlying diversity patterns at short and long-distance scales. These processes also appear pivotal to the proliferation of a drug resistance-associated multi-gene deletion on chromosome 31.

## Introduction

Species invasion creates unique opportunity for extreme evolutionary transformation. Small founding populations face unfamiliar selection pressures and sampling effects that drive genetic drift. Rapid changes in genetic makeup can occur and potentially dictate long-term population genetic structure throughout the invasive range^1^. Subsequent secondary introductions into the same area can also reshape diversity patterns in the population, e.g., by promoting introgressive hybridization events between ancestrally allopatric groups^2^. One medically relevant but little explored example of species invasion is represented by the introduction of *Leishmania infantum*, the parasitic agent of visceral leishmaniasis (VL), into the New World in conjunction with European colonization of the Americas beginning ca. 500 years ago^3,4^. Population structure and genetic change in *Leishmania* populations are of major concern to public health, as intra-specific genetic variation within this genus is associated with major differences in pathology^5–7^, drug resistance^8,9^, and other eco-epidemiological traits^10,11^. Driven in part by high karyotypic plasticity^12,13^, *Leishmania* parasites are capable of rapid adaptation and epidemic expansion after environmental change and/or bottleneck events^8^. Genetic recombination among *L. infantum* populations is another potential source of phenotypic diversity. Hybridization between divergent *Leishmania* isolates and species that cause distinct forms of disease^14^ can impact pathogenicity^14–16^, as well as facilitate vector^17^ and geographic range expansion^18^.

In the Americas, VL is a zoonosis caused by *L. infantum* infecting *Lutzomyia* sandflies, which have evolved in isolation of *Phlebotomus*, the Old World vector genus, for ca. 200 million years^19^. Domestic dogs represent principal reservoir hosts. The New World distribution of *L. infantum* now extends from the southern United States to northern Argentina^20^ and Uruguay^21^, but prevalence and/or reporting varies considerably across this range. Over 1000 VL cases have been recorded yearly in Brazil since the 1980s, first limited to the Northeast^22^ but now increasingly dispersed, including in urban areas such as those in Mato Grosso, Minas Gerais, and São Paulo state. VL infections are significantly less common elsewhere on the continent compared to Brazil^23^. Atypical cases, e.g., involving dermotropic or, more rarely, drug-resistant *L. infantum* isolates, are also sporadically observed in the New World^24–26^, but direct links between changes in disease progression and specific host or parasite factors are rarely established. A recently published genome-wide association study^27^, however, reports that *L. infantum* populations from Piauí, Maranhão, and Minas Gerais (Brazil) show resistance to miltefosine, an important anti-leishmanial drug, and associates this resistance to a large (>12 kb) deletion said to increase in prevalence from northern to southeastern Brazil (e.g., 5% in Rio Grande do Norte and 95% in Minas Gerais). The deletion is homozygous, spanning across all four copies of tetrasomic chromosome 31 (chr31). It covers four open reading frames as follows: LinJ.31.2370 (ecto-3′-nucleotidase/nuclease), LinJ.31.2380 (ecto-3’-nucleotidase precursor), LinJ.31.2390 (helicase-like protein), and LinJ.31.2400 (3,2-trans-enoyl-CoA isomerase). Ecto-3’-nucleotidases take part in purine salvage, macrophage infection, and escape from neutrophil extracellular traps^28–30^. Helicases are essential to DNA replication and 3,2-trans-enoyl-CoA isomerase contributes to fatty acid oxidation, a critical component of gluconeogenesis in amastigote parasite forms^31^. The simultaneous deletion of these four genes likely occurs through homologous recombination between repetitive elements bordering the deletion site^27,32^. The mechanisms by which the sub-chromosomal deletion has emerged in multiple different areas of Brazil, however, remain completely unknown. Its abundance and geographic distribution are also only rudimentarily described^27^. Analyses of demographic history, epidemiological phenotypes, and genetic covariation in deletion-carrying isolates are urgently required to clarify the emergence of the deletion genotype, quantify its spread, and understand implications for disease treatment and control.

The present study expands surveillance for the sub-chromosomal deletion into 17 states of Brazil, establishing that deletion-carrying isolates occur abundantly in both the country’s North and South. Although non-deletion genotypes appear common in the northern state of Piauí, a North-South gradient in chr31 deletion abundance (previously suggested to increase miltefosine treatment efficacy in Rio Grande do Norte^27^) does not occur in the dataset. Non-deletion type strains also appear common in the southwestern state of Mato Grosso do Sul. We go on to explore sequence diversity in 126 *L. infantum* genomes (59 newly sequenced in this study and 67 others from publicly archived datasets representing Brazil^27,33^, Honduras^34^, Panama^34^, Morocco^35^, and Europe^34^) in search of adaptive and/or demographic drivers of the widespread deletion genotype and discontinuous population structures in the New World. We describe phylogenetic relationships characteristic of one or few early ancestral mutant groups having risen to high prevalence by founder effect and observe a possible compensation for reduced ecto-3’-nucleotidase activity via increased ecto-ATPase activity in deletion-carrying isolates. We also demonstrate restored ecto-3’-nucleotidase activity in parasites with partial (heterozygous) sub-chromosomal deletion that clearly derive from natural mating between divergent deletion-carrying and non-deletion isolates. Several hybridizations appear to involve a secondary contact (SC) process in the West of Brazil but endogamic mating is also apparent in several states. Our results suggest a dynamic and yet incompletely charted distribution of *L. infantum* diversity in the New World. Volatile genotypes and biomarkers in this introduced range must be precisely monitored for effective disease control.

## Results

### High prevalence of multi-kilobase deletion on chr31

**Fig. 1:**
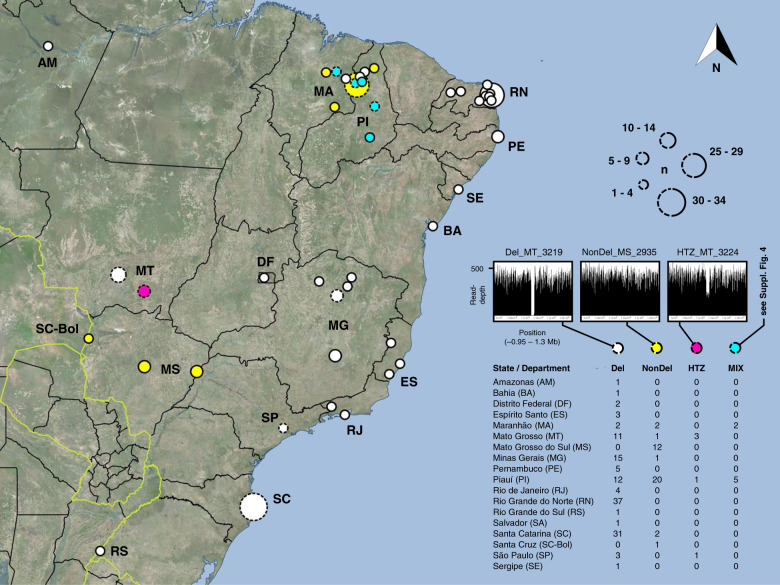
Different read-depth profiles found in *L. infantum* isolates from Brazil. Del isolates contain a >12 kb deletion between 1.122 Mb and 1.135 Mb on chr31 (e.g., Del_MT_3219 in the left graph). NonDel isolates do not contain the deletion, showing full read-depth at the locus (center graph). 8HTZ isolates are heterozygous for the deletion, with read-depth dropping to ca. 50% (right graph). Quantitative PCR confirmed heterozygosity at the deletion locus in monoclonal HTZ subcultures. MIX isolates appear to contain a mixture of NonDel and Del or HTZ profiles based on subclone PCR by Carnielli et al.^27^. However, full read-depth is observed at the deletion locus in all MIX isolates, except in MIX_PI_05A and MIX_PI_08A (showing ca. 75% read-depth, see Supplementary Fig. 4). This suggests that NonDel cells are more abundant than Del and/or HTZ cells within MIX isolates. Circle radius indicates the number of isolates (each from a different canine or human host) representing the study site. Dotted circles represent study sites where multiple read-depth profiles occur (see table inset). Fill color indicates the majority read-depth profile at such study sites. The map was created in the open-source geographic information system Quantum GIS version 2.18.4 using Open Layers plugin access to Bing Aerial imagery. Microsoft product screen shot(s) reprinted with permission from Microsoft Corporation.

Comparative analysis of 126 *L. infantum* genomes (19 from the Old World, 107 from the New World) and quantitative PCR (qPCR) screening of 75 additional New World samples (Supplementary Data 1) confirmed the occurrence of a >12 kb homozygous deletion on tetraploid chr31 (see somy values in Supplementary Fig. 1), previously described as a miltefosine sensitivity locus by Carnielli et al.^27^. The deletion occurred in samples from Brazil (126 of 177) and Honduras (2 of 2) but was absent from the Old World (0 of 19)^34^. New World samples without the deletion (referred to as “NonDel” as opposed to “Del” in subsequent whole-genome sequencing (WGS) analyses) were concentrated primarily in the Brazilian states of Piauí (20 of 38) and Mato Grosso do Sul (12 of 12) but they also occurred in Bolivia (1 of 1) and, as recently noted^34^, in Panama (2 of 2) (Supplementary Data 1 and Fig. 1). The deleted region spans chr31 base pair positions 1,122,848 to 1,135,161 in most Del samples (but see slight mapping variability within repetitive boundary regions in Supplementary Data 2) and comprises genes encoding for ecto-3’-nucleotidase (*LinJ.31.2370*), ecto-3’-nucleotidase precursor (*LinJ 31.2380*), helicase-like protein (*LinJ 31.2390*), and 3-2-trans-enoyl-CoA isomerase (*LinJ.31.2400*). Apart from these four genes, 38 coding regions showed significant copy number variation (CNV) between Del and New World NonDel groups in haploid somy estimate (s) comparison using Mann–Whitney *U* (MWU)-tests (Supplementary Data 3), but reassessment by analysis of covariance (ANCOVA) suggested that most of these differences are driven by population structure, i.e., common descent. Supplementary Fig. 2 illustrates how CNV profiles cluster by geographic origin, and geographic origin correlates to chr31 read-depth profile. The five coding regions, which remained significantly differentiated between Del and New World NonDel groups after controlling for geographic origin, encode amastin-like protein, nucleoside transporter, and paraflagellar rod protein paralogs (see asterisked columns in Supplementary Fig. 2). Effect size, however, is low (0.317 ≤ |∆*s* | ≤ 0.552) (Supplementary Data 3). We also did not note any substantial evidence for a direct relationship between the presence of deletion on chr31 and single-nucleotide polymorphism (SNP) or insertion-deletion variant (INDEL) differentiation among New World isolates (Supplementary Note 1, Supplementary Fig. 3, and Supplementary Data 4 and 5).

### Partial deletions occur via hybridizations between Del and NonDel isolates

**Fig. 2:**
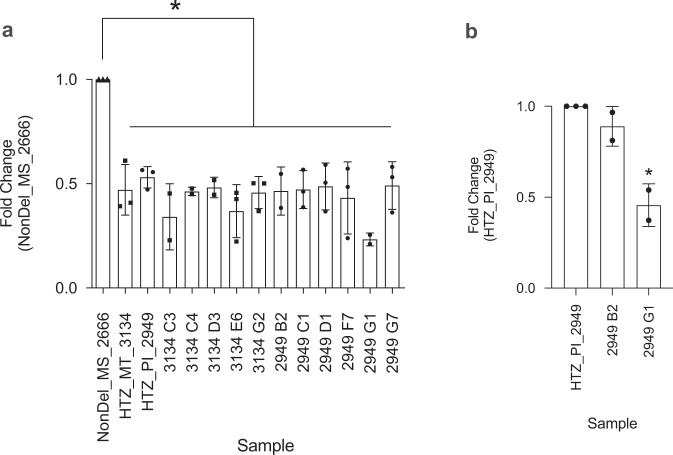
Quantitative PCR confirms that intermediate read-depth profiles represent heterozygous deletions in *L. infantum* clones. **a** HTZ_PI_2949 and HTZ_MT_3134 were selected as representatives of isolates for which read-depth drops to ca. 50% between 1.122 Mb and 1.135 Mb on chr31 (see Supplementary Fig. 4). DNA from monoclonal subcultures established from these two isolates was analyzed in qPCR targeting *LinJ.31.2380* (within the chr31 deletion site) and *LinJ.31.2330* (downstream of the chr31 deletion site). Differences in Ct values for *LinJ.31.2330* between each HTZ sample and the NonDel reference (NonDel_MS_2666) were used to normalize a fold change estimate at *LinJ.31.2380* based on the ∆∆Ct method by Livak and Schmittgen^75^. Student’s *t*-test was applied to test whether fold change estimates obtained from *n* = 3 independent reactions differed significantly from the 1 : 1 ratio represented by the reference sample. Results were considered significant at *p < 0.05 and indicate that intermediate read-depth profiles represent abundant heterozygous deletions as opposed to mixtures of deletion-carrying and non-deletion-type cells within isolates. **b** Fold change was calculated the same way for monoclonal HTZ subcultures using the parental isolate as the reference. Results indicate that “unbalanced” heterozygotes also occur, e.g., subclone 2949 G1 appears to contain three chromosome copies with the chr31 deletion and one copy without.

Six *L. infantum* samples sequenced in this study had an intermediate read-depth profile within the chr31 deletion site (Supplementary Data 1). In such genotypes, sequences mapped to the deletion site achieve ~50% read coverage relative to the rest of the chromosome (Supplementary Fig. 4), suggesting one of two scenarios: an abundance of cells with “equivalent” heterozygous sub-chromosomal deletion (i.e., cells in which two copies of chr31 carry the deletion and two copies do not) or an equally mixed population of Del and NonDel isolates. We therefore extracted DNA from 11 monoclonal subcultures established from two isolates representing putative heterozygotes (IOCL 2949 and 3134) and measured relative abundance of the deletion target by qPCR. Results from ten monoclonal subcultures showed a reduction of ca. 50% in the abundance of the amplified target sequence relative to the NonDel representative NonDel_MS_2666 (Fig. 2), confirming the presence of cells heterozygous at the deletion locus as opposed to a mix of (homozygous) Del and NonDel genotypes. Clone 2949 G1 showed 25% relative target amplification (Fig. 2b), suggesting the presence of three chromosome copies with the deletion and one copy without. Subpopulations with different levels of heterozygosity appear to occur but equivalent heterozygotes—i.e., cells in which two copies of chr31 carry the deletion and two copies do not—appear most abundant based on read-depths from parental culture sequencing (Supplementary Fig. 4). Aside from these six isolates (hereafter termed “HTZ”), seven isolates sequenced by Carnielli et al.^27^ simultaneously showed Del and NonDel deletion site PCR amplicons in the previous study but ca. 80–100% read-depth within the deletion site (Supplementary Fig. 4). We refer to these samples as “MIX” without resolving the extent to which their sequence reads represent mixed-strain or monoclonal cell populations.

Given the vast geographic range occupied by Del isolates (Fig. 1), we considered the possibility of independent deletion emergence as an adaptive process recurring frequently across the American continent. Under such scenario, HTZ isolates might represent former NonDel genotypes currently in transition to the homozygous (i.e., complete, fourfold) deletion state. This NonDel to Del transition might occur via successive independent locus deletion on different chromosome copies or via locus deletion on a single chromosome copy followed by over-replication of the deletion-containing copy and under-replication of non-deletion copies during mitosis. Following ADMIXTURE analysis (Supplementary Fig. 5), however, in which HTZ_MT_3134, HTZ_MT_3135, HTZ_MT_3137, HTZ_MT_3224, and HTZ_SP_3254 (i.e., all HTZ samples, except HTZ_PI_2949) received simultaneous Del + NonDel group assignment, we also considered the alternate hypothesis that HTZ isolates represent hybrid offspring forming at contact zones between Del and NonDel groups (Fig. 1). Support for this alternate hypothesis quickly accumulated through several analyses and metrics.

**Fig. 3:**
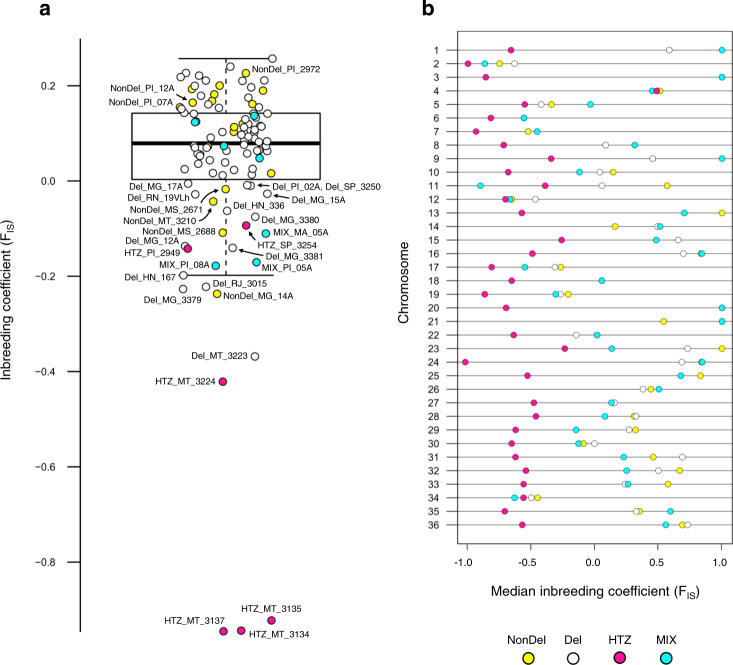
Homozygosity relative to Hardy–Weinberg expectations in New and Old World *L. infantum* isolates. **a** The box plot shows median and interquartile ranges of genome-wide inbreeding coefficients (F_IS_). Values are generally high for New World isolates. Values for HTZ isolates, however, all occur below the second quartile and strong excess heterozygosity is suggested in HTZ_MT_3134, HTZ_MT_3135, and HTZ_MT_3137. **b** Relatively low genome-wide F_IS_ in HTZ isolates is not driven by values from a subset of chromosomes. Values appear low throughout the genome. Circle fill color indicates New vs. Old World origin and read-depth profile on chr31.

**Fig. 4:**
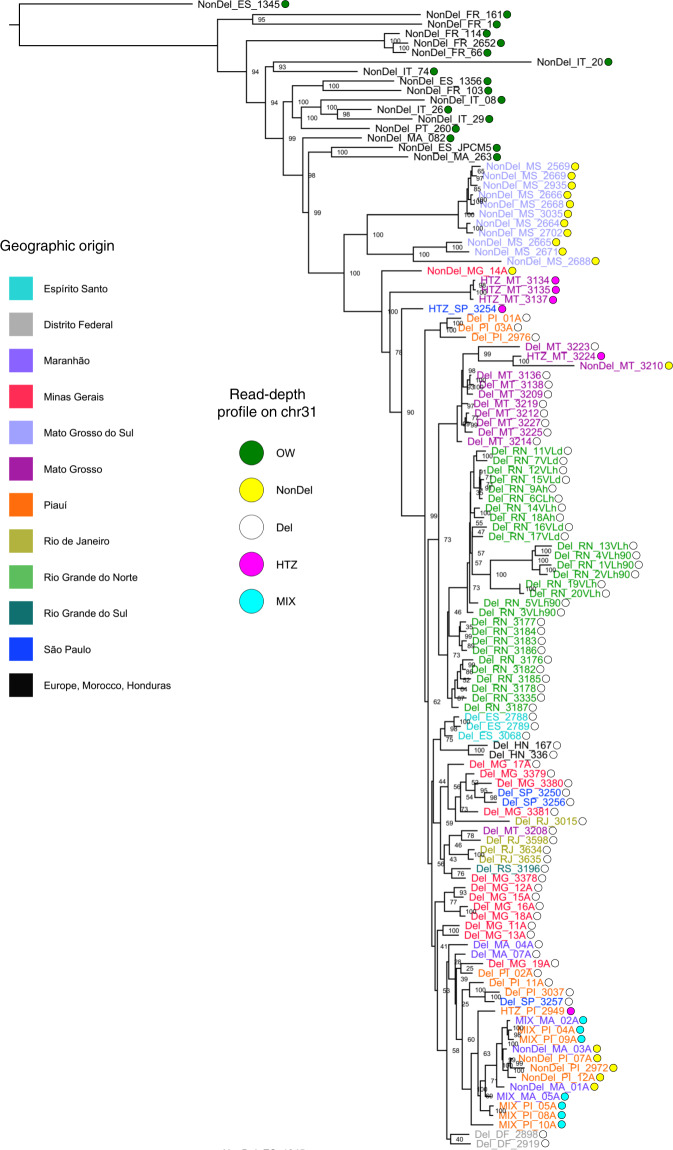
Phylogenetic relationships among New and Old World *L. infantum* isolates. The maximum-likelihood tree was built using a general time-reversible substitution model with branch lengths corrected for ascertainment bias (i.e., the use of only nonvariant sites in sequence alignment). Pairwise genetic distances are haplotype-based, defined as the proportion of non-shared alleles across all SNP sites for which genotypes are called for all individuals (i.e., no missing data in alignment). Outlier isolates NonDel_MS_MAM, NonDel_FR_47, NonDel_PT_151, NonDel_PA_317, and NonDel_PA_85 are excluded. *L. donovani* strain MHOM/NP/03/BPK282/0 was temporarily included as an outgroup, to identify an *L. infantum* sample to subsequently root the tree. NonDel_ES_1345 became the outgroup. Circle fill color indicates New vs. Old World origin and read-depth profile on chr31. Font color specifies states sampled in Brazil. Isolates from other countries are labeled in black.

**Fig. 5:**
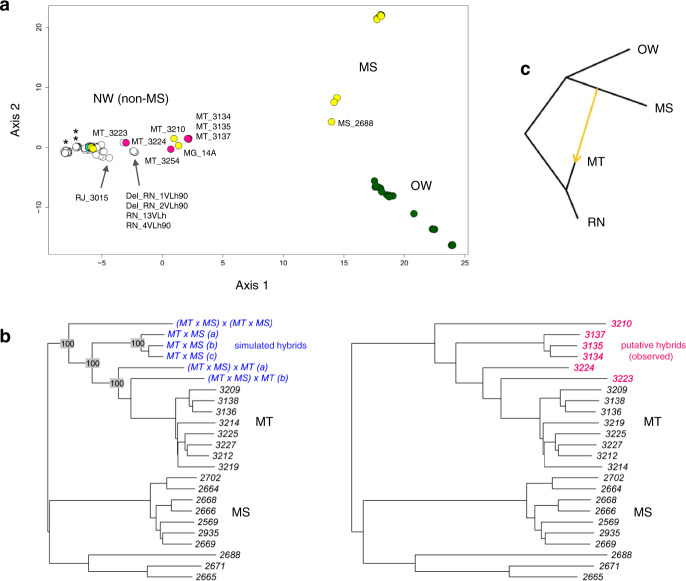
Metric multidimensional scaling, simulated mating, and tree-to-graph conversion suggest admixture and hybridization between Del and NonDel *L. infantum* groups. **a** Metric multidimensional scaling separates New and Old World (NW and OW) isolates on two axes of variation (goodness-of-fit = 0.40). NonDel isolates from Mato Grosso do Sul (MS) and Del isolates from Rio Grande do Norte (RN, see asterisk) and Mato Grosso (MT, see double-asterisk) position at opposite ends of axis 1, the primary axis of divergence within and between NW populations. HTZ isolates occur at intermediate positions (see pink circles) between these dissimilar groups. Other isolates with such intermediate positions are labeled and may also represent mating events between dissimilar groups. Gray, white, and cyan fill colors, respectively, indicate NonDel, Del, and MIX read-depth profiles found in the NW. Circles for OW (NonDel) isolates are green. Five outlier isolates are excluded as in Fig. 4. **b** Neighbor-joining positions of simulated hybrids (blue font, left tree) correspond to those of observed HTZ isolates (pink font, right tree) from MT. Hybrids were simulated in two steps. Random 50% haplotype contributions were first drawn from Del and NonDel isolates observed in MT and MS. The resultant offspring genotypes were then either let diversify through random mutation or subjected to a second round of Mendelian recombination as before. The same tree topology resulted in each of 100 simulation replicates. Trees are midpoint-rooted as opposed to outgroup-rooted as in Fig. 4. **c** Given that mating can create non-treelike divergence patterns within species, TreeMix^66^ was used to search iteratively for up to five migration edges that improve the fit of a maximum-likelihood tree built based on Gaussian approximation of genetic drift among isolates from MT, MS, RN, and OW groups. This input tree (black edges) suggests dichotomous differentiation into MT/RN and MS/OW clades and has a log-likelihood of 84.9206. Tree-to-graph conversion by addition of a migration edge from MS to MT increases log-likelihood to 84.9775. No other edges further increase the fit of the input tree. A four-population test^76^ also supports post-split admixture between MS and MT or RN, because differences in allele frequencies between MT and RN isolates correlate with those within the other population pair (F_4_-statistic = 5 × 10^−5^, *Z*-score = 3.51).

HTZ samples showed marked, statistically significant reductions in total homozygosity and F_IS_ values (which describe the extent to which individual heterozygosity is reduced by inbreeding) relative to Del and to New World NonDel isolates (Fig. 3a and Supplementary Data 6). Median F_IS_ was lowest in HTZs (relative to Del and New World NonDel groups) in 33 of 36 chromosomes (Fig. 3b). Except for HTZ_PI_2949, HTZs occurred in peripheral positions relative to monophyletic Del subclades in maximum-likelihood phylogeny (Fig. 4) and showed intermediate positions on PCoA axis 1 (Fig. 5a). We also constructed neighbor-joining trees from phased chromosomes (Supplementary Fig. 6) and homologous haplotypes of Mato Grosso HTZ isolates generally divided between Mato Grosso Del and Mato Grosso do Sul NonDel clades (i.e., one HTZ haplotype appearing similar to both haplotypes of Mato Grosso Del isolates and the other HTZ haplotype appearing more similar to both haplotypes of NonDel isolates from Mato Grosso do Sul). This divided HTZ haplotype clustering suggested a process of nuclear genetic exchange in which homologous chromosomes from distinct progenitors are found within hybrid offspring, consistent with sexual mating or, less parsimoniously (because ploidy levels did not appear aberrant (Supplementary Fig. 1)), genome fusion events. F_ST_ differentiation to Mato Grosso do Sul samples also fluctuated among HTZ chromosomes, consistent with chromosomal reassortment as a result of mating between Del and NonDel isolates (Supplementary Fig. 7). We further examined a potential hybrid origin by comparing the phylogenetic positions of HTZ isolates from Mato Grosso with those generated by simulated sexual mating (see Supplementary Codes^36^) between populations from Mato Grosso and nearby Mato Grosso do Sul. Phylogenetic positions for simulated hybrids corresponded to those observed for HTZ isolates (Fig. 5b). In these simulations, we also hypothesized the presence of second-generation (F_2_) hybrids, i.e., we simulated backcrossing and hybrid inter-crossing to account for the origin of Mato Grosso samples Del_MT_3223 and NonDel_MT_3210 (respectively). These two samples are not heterozygous for the deletion on chr31 but show low genome-wide F_IS_ (Fig. 3a) and place near HTZ samples in Principal Coordinates Analysis  (PCoA) (Fig. 5a). Phylogenetic positions of the simulated F_2_ hybrids matched those observed for Del_MT_3223 and NonDel_MT_3210. Similar F_2_ hybridization events may also explain the outlying phylogenetic positions of samples such as NonDel_MG_14A or NonDel_MS_2688 (Figs. 4 and 5a, and Supplementary Fig. 6).

### Demographic drivers of patchy population structure and hybridization events

The *L. infantum* group from Mato Grosso do Sul stood out in above analyses given its complete lack of Del genotypes and its basal phylogenetic position relative to all other New World isolates (Fig. 4). This outgroup also showed higher nucleotide diversity (π) per site (0.046 vs. 0.061, respectively), more than twice as many private SNP sites per sample (15.3 vs. 31.8) and lower F_ST_-differentiation to Old World isolates (0.413 vs. 0.303) than did the rest of the New World sample set (Table 1).

**Table 1:** Population genetic descriptive metrics for New World and Old World *L. infantum* groups.

Group (*n*)	*K*	Het.	PS	PRS	*π*	F_ST_ to OW	F_ST_ to MS	F_ST_ to non-MS
Non-MS (80)	2.01	0.122	1782	15.3	0.046	0.419	0.495	0.000
MS (11)	2.00	0.324	903	31.8	0.061	0.304	0.000	0.495
Old World (17)	2.00	0.195	3069	149.1	0.125	0.000	0.304	0.419

*F*_*ST*_ between-group fixation index, *Het.* mean heterozygosity, *K* mean number of alleles per locus, *MS* Mato Grosso Do Sul, *n* sample size, *non-MS* New World, excluding MS, *PRS* private sites, per sample, *PS* total polymorphic sites, *π* nucleotide diversity.

HTZ and MIX genotypes are not used in this analysis.

We used a pattern-process modeling approach to better understand the divergence history of the MS group and its paths to contemporary admixture with Mato Grosso isolates. We considered a “SC” model of divergence, in which Mato Grosso do Sul parasites diverged in isolation from Mato Grosso parasites but later reestablished gene flow, perhaps due to separate introductions from the Old World into Brazil. Alternatively, Mato Grosso do Sul and Mato Grosso groups may have followed an “isolation with migration” (IM) model of divergence, whereby gene flow between them never fully ceased but Mato Grosso do Sul parasites underwent significant divergence due to local selection pressures or secondary bottleneck events. For both SC and IM models, we simulated individual genome-wide SNP diversity in three variations relating to bottleneck (yes/no in Mato Grosso founder population), admixture type (hard introgression and/or permanent migration vs. temporary genetic exchange), and rate of gene flow (constant or variable over time). We also ran simulations for two implausible models of Mato Grosso do Sul—Mato Grosso divergence, “strict isolation” (SI, i.e., gene flow between the two populations permanently ceased) and “ancient migration” (AM, i.e., gene flow between the two populations permanently ceased following an early period of continuous gene flow). These served as “negative” controls for the Approximate Bayesian Computation via Random Forests (ABCRF)^37^ method, which uses random forests to rank the fit of observed vs. simulated summary statistics. Simulations for Mato Grosso do Sul—Old World and Mato Grosso—Old World population pairs, both assumed to follow an AM with bottleneck (AM_bot_) model of divergence, provided additional “positive” controls (see fastsimcoal2^38^ template files and model illustrations in Supplementary Data 7, see Supplementary Codes^36^, and Supplementary Fig. 8). Following expectations, the AM_bot_ model achieved highest support for both Mato Grosso do Sul—Old World and Mato Grosso—Old World divergence (Table 2). Also as expected, AM and SI models received lowest (near zero) support for the Mato Grosso do Sul—Mato Grosso population pair. Support was highest for the SC base model (350 of 1000 votes, Table 2), with subsequent parameter optimization specifying slightly higher gene flow (Migration - MIG) towards Mato Grosso than towards Mato Grosso do Sul (Table 2) as also previously indicated by tree-to-graph optimization and F_4_-statistics (Fig. 5c). The IM_change_ model, which suggests that rate of gene flow between Mato Grosso do Sul and Mato Grosso changed but never fully ceased over time, received the second-highest support (253 votes). Overall posterior probability (0.504), however, was low due to the inclusion of two highly similar additional variants of each IM and SC base model in analysis (Table 2). We therefore re-ran the ABCRF process using only the four base models SC, IM, SI, and AM. In this analysis, the SC model achieved a much clearer majority (627 votes) over IM (264), SI (65), and AM (44), and posterior probability rose to 61%. Taken together, the above analyses suggest that hybrid genotypes observed in Mato Grosso involve a SC process after the bottlenecking of *L. infantum* from the Old World into Brazil. It remains to be established, however, if Mato Grosso do Sul and Mato Grosso parasites temporarily diverged in isolation due to independent importations or whether temporary isolation began after common introduction to the New World.

**Table 2:** Demographic simulation in fastsimcoal2 and model selection by Approximate Bayesian Computation via Random Forests (ABCRF).

	Model	Pop. 1/Pop. 2	CV	*N*_draws_	
Models of divergence between MT and MS *L. infantum* groups	AM	MT/MS	0.035	474,177	
	IMbot	MT/MS	0.086	452,533	
	IM_change_	MT/MS	0.243	476,483	
	IM	MT/MS	0.085	474,263	
	SC	MT/MS	0.350	473,082	*Selected model
	SCbot_nomig_	MT/MS	0.109	427,249	
	SC_nomig_	MT/MS	0.078	474,782	
	SI	MT/MS	0.014	466,136	
	PP = 0.504				
	MIG_MT>>>MS_ = 0.254				
	MIG_MS>>>MT_ = 0.300				
Models of divergence between MT and OW *L. infantum* groups	AMbot	MT/OW	0.304	432,323	*Selected model
	AM	MT/OW	0.186	458,125	
	IM_change_	MT/OW	0.106	470,330	
	IM	MT/OW	0.215	459,566	
	SC	MT/OW	0.161	421,405	
	SC_nomig_	MT/OW	0.013	464,907	
	SIbot	MT/OW	0.003	385,170	
	SI	MT/OW	0.012	409,244	
	PP = 0.485				
	FOU = 0.204				
Models of divergence between MS and OW *L. infantum* groups	AMbot	MS/OW	0.385	413,704	*Selected model
	AM	MS/OW	0.161	472,457	
	IM_change_	MS/OW	0.145	473,388	
	IM	MS/OW	0.170	471,073	
	SC	MS/OW	0.025	471,677	
	SC_nomig_	MS/OW	0.031	472,251	
	SIbot	MS/OW	0.035	463,789	
	SI	MS/OW	0.048	457,084	
	PP = 0.521				
	FOU = 0.292				

*CV* classification vote, i.e., the number of times a model is selected in a forest of 1000 trees (the model with the most votes corresponds to the model best suited to the dataset), *FOU* bottleneck size, i.e., the fraction of prior population size at the end of the bottleneck, *MIG*_*x»>y*_ migration rate from *x* to *y*, *MS* Mato Grosso do Sul, *MT* Mato Grosso, *N*_*draws*_ number of parameter draws simulated by fastsimcoal2 as input for ABCRF, *OW* Old World, *Pop.* population, *PP* ABCRF approximation of the posterior probability of the selected model.

In fastsimcoal2 simulation, values for past and present population sizes were drawn randomly from a uniform distribution between 100 and 106 individuals. Values for time of secondary contact were drawn randomly from a uniform distribution between 0 and 2 × 10^4^ generations before present. Values for relative migration rates between populations were drawn randomly from a log-uniform distribution between 10^−10^ and 0.1. Values for bottleneck size were drawn randomly from a uniform distribution between 0.05 and 0.5. The mutation rate was fixed at 1.99 × 10^−9^ mutations per bp on all chromosomes. The ten different demographic models are illustrated in Supplementary Fig. 8 and template file content is provided is provided in Supplementary Data 7 at Zenodo^36^.

### Introgression disrupts monophyletic ancestry of Del isolates

In light of the ample evidence for frequent hybridization described above, but also positive inbreeding coefficients in most samples (Fig. 3a), we wondered whether mating between more similar Del and NonDel genotypes likewise occurs frequently in Brazil. This possibility appeared especially relevant in the case of Piauí and Maranhão isolates NonDel_MA_01A, NonDel_MA_03A, NonDel_PI_07A, NonDel_PI_12A, and NonDel_PI_2972, as these were the only NonDel isolates which nested within what otherwise appeared as a Del-exclusive, monophyletic clade (Fig. 4). NonDel_PI_2972 featured the highest genome-wide F_IS_ value of any NonDel sample (Fig. 3a), and NonDel_MA_03A, NonDel_PI_07A and NonDel_PI_12A shared the dataset’s highest F_IS_ value for chr31 (F_IS_ = 0.92). Of all large (>1 Mb) chromosomes (i.e., chromosomes 26 to 36), chr31 ranked highest in F_IS_ for these 3 samples and for NonDel_MA_01A (F_IS_ = 0.85). For all other samples, the median rank of F_IS_ for chr31 relative to other large chromosomes was 3.

We also noted that F_ST_ between the nested NonDel clade and phylogenetically similar Del isolates increased specifically on chr31 (Supplementary Fig. 7), showing higher values only on the (small) chromosomes 3 and 16 and near zero (i.e., no differentiation) on most other chromosomes. This subtle chr31-specific divergence from Del isolates was not statistically significant (Tukey and Kramer (Nemenyi) test) but was substantiated by analysis focused on “Del-distinctive” sites, i.e., sites at which >90% of Del isolates and <50% of New World NonDel isolates show non-reference genotypes. Of 470 such Del-distinctive sites, 466 also showed a non-reference genotype in at least one member of the nested NonDel group. The remaining 4 sites, however, all occurred on chr31 (nucleotide positions 387,322, 423,106, 1,254,441, and 1,390,994). Chr31-specific divergence was also exposed by running ADMIXTURE analysis at *k* = 2 separately for all large chromosomes in New World isolates, excluding the putatively outcrossing groups Mato Grosso and Mato Grosso do Sul. The cumulative total of all individual Del ancestry proportions matching the Piauí/Maranhão NonDel population assignment was only 15% for chr31 as opposed to 61% for chr26, 56% for chr27, 81% for chr28, 54% for chr29, 75% for chr30, 71% for chr32, 74% for chr33, 66% for chr34, 46% for chr35, and 37% for chr36.

These observations are consistent with the hypothesis that the nesting of Piauí/Maranhão NonDel isolates in Fig. 4 represents an admixture process between Del isolates and closely related NonDel isolates, whereby introgression of polymorphisms on chr31 may be preserved more so than on other chromosomes during subsequent backcrossing or mitotic haplotype selection events^13^. This possibility of introgression perturbing Del monophyly is also supported by evaluating the likelihood of trees constructed under a constraint that forces Piauí/Maranhão NonDel isolates to group outside of the Del clade, specifically, applying newick constraint = ((Piauí/Maranhão NonDel isolates, Mato Grosso do Sul NonDel isolates), (all Del isolates)). The Shimodaira–Hasegawa test^39^ suggests that maximum likelihood resulting from such constrained tree construction is not significantly lower than that of Fig. 4’s unconstrained maximum-likelihood tree (*p* = 0.431). A constraint forcing Honduran Del samples out of the Del clade, by contrast, does result in a significantly less likely tree (*p* = 0.045), further substantiating the geographically widespread expansion of a monophyletic Del clade. Nevertheless, we cannot conclude definitively whether the deletion found in all isolates of this clade stems from a single ancestral mutant lineage or if multiple, closely related ancestral lineages experienced separate deletion events (see Supplementary Notes 2 and 3, which describe a phylogenetic signal on deletion stop site coordinates listed in Supplementary Data 2; although stop site variation is minimal, its phylogenetic signal raises the possibility that distinct deletion mutations or post-deletion modifications occurred among progenitors the Del clade).

### Phenotypic consequences of the sub-chromosomal deletion

**Fig. 6:**
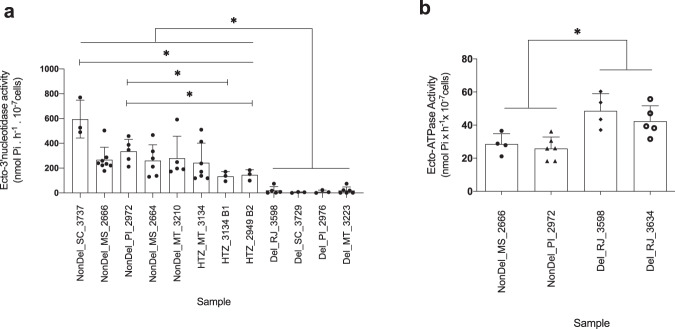
Ecto-3’-nucleotidase and ecto-ATPase activity correlates to read-depth profiles on chr31. **a** Ecto-3′-nucleotidase activity was quantified by measuring the rate of inorganic phosphate (_Pi_) release during adenosine 3’-AMP hydrolysis as described in Freitas-Mesquita et al.^28^. Bar plots show mean and S.E. for at least three replicate assays (*n* = 3 independent experiments). Welch’s *t*-test was applied to test for statistical significance between pairs of samples at *p < 0.05. Results indicate a significant reduction of enzymatic activity in Del isolates relative to all NonDel and HTZ isolates. Activity appears significantly higher in NonDel_SC_3737 than in other NonDel and HTZ samples. Activity also appears significantly higher in NonDel_PI_2972 relative to HTZ_3134_B1 and HTZ_2949_B2 subcultures. **b** Ecto-ATPase activity was quantified with the same protocol except replacing 3’-AMP with equimolar ATP and Mg^2+^. *T*-tests between NonDel and Del isolates suggest higher ecto-ATPase activity in Del isolates than in all NonDel isolates, but larger samples sizes are required to substantiate the effect.

Finally, we performed an assay for ecto-3’-nucleotidase activity (Fig. 6a) in Del, NonDel, and HTZ samples representing different levels of phylogenetic similarity and various states of Brazil. Results demonstrate heavily reduced ecto-3’-nucleotidase activity in Del isolates relative to HTZ and NonDel isolates (*p* < 0.05) (despite no polymorphisms observed in an ecto-3’-nucleotidase paralogue present on chr12). Inter-individual variation in ecto-3’-nucleotidase activity also occurred among NonDel and HTZ samples: NonDel_SC_3737 showed significantly higher activity than all other NonDel and HTZ samples (*p* < 0.05) and NonDel_PI_2972 showed significantly higher activity than monoclonal HTZ subcultures HTZ_3134_B1 and HTZ_2949_B2 (*p* < 0.05). Activity in these two subcultures did not significantly differ to that in the uncloned HTZ isolate HTZ_MT_3134 (*p* < 0.05). We also measured the activity of ecto-ATPase (an enzyme thought to be involved in purine salvage pathways^40,41^ independent of ecto-3’-nucleotidase) in Del and NonDel isolates (Fig. 6b). Higher ecto-ATPase activity occurred in Del isolates than in NonDel isolates (*p* < 0.05), suggesting the possibility that alternative molecular pathways compensate effects of ecto-3’-nucleotidase deletion on chr31.

## Discussion

Our results reveal the widespread distribution of a major genetic alteration found in New World *L. infantum* isolates, clarifying that a four-gene deletion on chr31 predominates in southeastern, eastern, and (unlike previously suggested^27^) northeastern Brazil. In addition to two Panamanian non-MON-1 samples that do not show this drug resistance-associated mutation^34^, a divergent non-deletion group occurs in the southwestern Brazilian state of Mato Grosso do Sul. Coalescence modeling suggests a SC process between these divergent NonDel parasites and members of the widespread, likely monophyletic, Del clade. This admixture involves genome-wide hybridization events that transfer Del chr31 homologs into paraphyletic NonDel groups. Genetic exchange between closely related Del and NonDel strains, followed by further inbreeding and/or mitotic haplotype selection^13^, may also explain the presence of NonDel isolates with high homozygosity and low (chromosome-specific) divergence to Del strains in other regions of Brazil, e.g., in Piauí and Maranhão.

The extensive evidence of genetic exchange we describe substantiates the importance of this process in trypanosomatid evolution. We observed a direct effect of hybridization on phenotype, showing substantially higher activity of the potential virulence factor ecto-3’-nucleotidase^40^ in putative F_1_ hybrids relative to parental Del genotypes. Changes to human pathogenicity are also directly implied by the association between locus deletion and miltefosine treatment efficacy suggested by Carnielli et al.^27^. Our observations add to a growing body of evidence that genetic exchange plays an important role in the spread of epidemiologically relevant traits through natural trypanosomatid populations (e.g., drug resistance in *Leishmania donovani*^9^, vector infectivity in *Leishmania major*/*L. infantum*^17^, and human infectivity in *Trypanosoma brucei*^42,43^) and are consistent with a “mixed mating model of reproduction”^44^ in the *Leishmania* genus. Similar to observations in *Trypanosoma cruzi*^45^, different rates of sex and clonality may occur in *L. infantum* depending on demographic or ecological variation within landscapes or between the parasite’s evolutionarily native (Old World) and introduced (New World) range. Our study suggests that hybridization occurs frequently at SC zones as well as the possibility that mating is generally common, and perhaps advantageous, in non-native and/or bottlenecked groups. *Plasmodium* parasites, e.g., have been suggested to alter sex allocation and inbreeding rates to enhance success in the mosquito vector, with rates dependent on the diversity of sympatric strains^46,47^.

This study also substantiates that CNV is a highly heritable form of polymorphism in the *Leishmania* genus^48^. In genome-wide read-depth analysis excluding the focal deletion on chr31, CNV-based hierarchical clustering mirrored the SNP-based phylogeny and geographical origins of the sample set, suggesting that baseline gene copy numbers or deletion/amplification programs triggered in vitro are conserved among related isolates. Results do not suggest that any single CNV regime underlies enzymatic changes (e.g., ecto-ATPase upregulation) that might be occurring to compensate loss of function within the deleted locus on chr31. Such compensation may occur through unique (i.e., sample specific) CNV solutions or by various other epigenetic, post-transcriptional or posttranslational effects. The five copy number differences showing statistical significance between Del and New World NonDel groups do nevertheless deserve further investigation. Effect sizes were small but the transport (*LinJ.08.0700*, *LinJ.15.1240*, *LinJ.15.1250*) and cytoskeletal (*LinJ.29.1880*, *LinJ.29.1890*) proteins involved carry out vital cell functions, variation in which has also been linked to drug resistance in previous research^49–51^.

Taken together, this study highlights the pivotal roles and interplay of genetic exchange and demographic history in shaping *L. infantum* sequence and karyotypic diversity in the New World. Although the importance of genetic exchange has been increasingly appreciated in recent years, the consequences of post-Columbian range expansion in this species are seldom acknowledged and remain poorly understood. The process is often simplified as yielding a low-diversity, homogeneous *L. infantum* population, one which might display clinical variation due to environmental or host-related factors but less likely due to parasite genetic traits^52^. Our observations reject such scenario and open up many new questions on the different sources and yet unknown extent of *L. infantum* diversity in the New World. For example, could distinct local selection pressures (e.g., the presence of *Lutzomyia* *cruzi* as opposed to *Lutzomyia longipalpis*) contribute to the success of a separately introduced parasite population in Mato Grosso do Sul? Might this population, and other unobserved populations, involve importations from different colonial empires, e.g., the French or Spanish, aside from the Portuguese^53^? Does the widespread distribution of Del genotypes in the Americas reflect fitness advantages within the parasite’s narrow host/vector spectrum in the New World? Could reduced virulence as a consequence of reduced ecto-3’-nucleotidase activity play a role in the predominance of Del strains? Alternatively, is locus deletion neutral or deleterious, and the widespread distribution of deletion-carrying strains is principally an allele surfing effect (a phenomenon in which new mutations spread rapidly across new territories due to their emergence on an expanding wave front, where population density is low and growth rates are high^54^)? We hope that this study inspires much new research on such questions and recommend greater attention to complex *L. infantum* population structure and hidden genetic diversity in future disease control.

## Methods

### Parasite samples and whole-genome sequencing

All 201 *L. infantum* samples assessed in this study are listed in Supplementary Data 1, which also provides information on alternative nomenclatures, geographic origin, chr31 read-depth profile (i.e., whether or not isolates carry the sub-chromosomal deletion described by Carnielli et al.^27^) and analysis type (i.e., WGS analysis or quantitative real-time PCR). All 59 *L. infanum* strains sequenced in this study were obtained from the Coleção de *Leishmania* da Fundação Oswaldo Cruz (CLIOC). In all cases *Leishmania* were isolated from patients as part of normal diagnosis and treatment with no unnecessary invasive procedures and with written and/or verbal consent recorded at the time of clinical examination. All strains were cultured in biphasic (Novy–MacNeal–Nicolle (NNN) + Schneider’s) medium prior to genomic DNA extraction (DNeasy Blood & Tissue Kit (Qiagen). Fragmented DNA (mean insert size = 377 nt) was sequenced using Illumina NextSeq 500 and HiSeq 2500 instruments, and mapped to the MCAN/ES/98/LLM-724 (termed JPCM5 elsewhere in the text) reference assembly available at https://tritrypdb.org/common/downloads/release-33/LinfantumJPCM5/fasta/ using default settings for BWA-mem v0.7.3^55^. Publicly archived and/or previously published *L. infantum* reads^27,33–35^ were mapped using the same conditions as the newly generated WGS data (see mapping coverage per sample in Supplementary Data 1). For enzymatic assays (see below), parasites were cultivated in flasks containing Schneider’s medium with 20% fetal calf serum (FCS) and 2% filtered urine until late log-phase expansion. Growth curves were obtained to rule out samples with possible confounding differences in replication rate. All parasites used in the experiments showed similar replication rates. These parasites had been kept in culture between 10 and 20 passages after isolation and cryopreservation by CLIOC.

### Phylogenetic, demographic modeling, and selection analyses

SNPs and INDELs were called using population-based genotype and likelihood assignment in Genome Analysis Toolkit (GATK) v3.7.0^56^ (programs “HaplotypeCaller” and “GenotypeGVCFs”). We excluded tightly clustered variants (i.e., more than three SNPs or INDELs within ten bases) as well as those achieving <1500 phred-scaled call quality (QUAL as calculated by GATK). We also excluded variants detected in non-unique mapping positions of the reference genome. Specifically, we generated synthetic, non-overlapping 125 nt sequence reads from the JPCM5 reference assembly (excluding unassigned contigs) and mapped these reads back to this same assembly using the “mappability” program in the Genomic Multi-tool software suite v1.376^57,58^. Only variants from areas with perfect synthetic mapping coverage were retained. The above filtering decisions were guided by results for the JPMC5 reference strain (re-sequenced in this study using paired-end 2 × 150 nt Illumina NextSeq).

We visualized genome-wide phylogenetic relationships among samples by maximum-likelihood tree construction in IQ-Tree v1.5.4^59^, optimizing a general time-reversible substitution model based on single-nucleotide differences at polymorphic sites. The pseudo-sequence alignment used as input for IQ-tree was generated without phasing, assuming allele order as reference allele first and alternate allele second at heterozygous sites. *L. donovani* isolate MHOM/NP/03/BPK282/0 was temporarily included as an outgroup in order to root the tree. Five samples were excluded from the tree and all other SNP/INDEL-based analyses due to radical divergence from the rest of the sample set. These were NonDel_MS_MAM (likely a mixture of divergent strains^34^), NonDel_PA_317 and NonDel_PA_85 (both non-MON-1^34^), and NonDel_FR_47 and NonDel_PT_151 (reason for divergence unclarified). Euclidean dissimilarities among genotypes were visualized by metric multidimensional scaling (PCoA)^60^ using the base “stats” package v3.4.1 in R v3.4.1^61^. Ancestry estimation was performed using ADMIXTURE v1.3^62^ (using all SNPs for which genotypes were called in all individuals) and putative first-generation (F_1_) hybrid genotypes simulated from observed data by calculating allele frequencies of two parental populations, then drawing gametes following a multinomial distribution in the R package “adegenet”^63^. Second-generation (F_2_) hybrids were simulated by iterating the same process but with parental populations comprising the prior F_1_ genotypes. Neighbor-joining (NJ) relationships based on a Euclidian distance matrix of alternate allele counts (i.e., 0 = homozygous reference, 1 = heterozygous reference/non-reference, and 2 = homozygous non-reference) were plotted for the simulated and observed data with the ‘ape’ package v5.0^64^ in R v3.4.1^61^ (see Supplementary Codes^36^). For haplotype-based NJ trees, heterozygous SNPs were computationally phased over 30 iterations using BEAGLE v4.1^65^. Manual verifications are provided in the Supplementary Information. We tested for admixture events in populations showing poor fit (high residuals) in tree-based phylogenies by searching non-treelike (graph) structures for higher maximum-likelihood in TreeMix v1.13^66^. The program also implements F_4_-statistics to test significance of the improved fit.

Demographic histories inferred from phylogenetic analyses above were further tested by simulating ten different scenarios of pairwise divergence (AM; AM with bottleneck, isolation with (constant) migration; isolation with (constant) migration and bottleneck; isolation with change in migration; SC; SC without hard admixture; SC without hard admixture with bottleneck; strict isolation; and strict isolation with bottleneck) and associated genome-wide SNP polymorphism in fastsimcoal2 v2.5.2^38^. For each of >100,000 random parameter sets simulated per divergence model, 12 summary statistics (total number of polymorphic sites; mean total heterozygosity; number of segregating sites per population; number of private sites per population; number of pairwise differences per population; mean and SD of segregating sites over populations; and mean and SD of pairwise differences over populations) were computed in ARLSUMSTAT v3.5.2^67^. Model selection and parameter estimations followed by ABCRF using 1000-tree regression forests in the “abcrf” package v1.7^37^ in R v3.4.1^61^. Observed genotypes of putative F_1_ and F_2_ hybrids were not included in the calculation of summary statistics for modeling divergence between New World groups.

Selection analyses between predefined groups (deletion-carrying and non-deletion type isolates) were performed by assessing site-wise F_ST_ neutrality with BayeScan v2.1^68^. We set prior odds for the neutral model to 100 and retained loci with log_10_ *q*-values < −2, where false discovery rate is expected to fall below 1%. Results were then filtered for coding regions and SNP and INDEL effects predicted with SNPEff v3t^69^ using the JPCM5 annotation file available at https://tritrypdb.org/common/downloads/release-33/LinfantumJPCM5/gff/data/.

### Chromosomal and gene copy number analyses

To estimate chromosomal somy, we calculated mean-read-depth (m) for successive 1 kb windows using SAMtools v0.1.18^70^ “depth” (default options) and then calculated a “median-of-means” (*M*_m_) for each chromosome. We let the 40th percentile (p40) of *M*_m_ values represent expectations for the disomic state, estimating copy number for each chromosome by dividing its *M*_m_ by the sample’s p40 value and multiplying by two. Copy numbers were then visualized with the “heatmap.2” function in the “gplots” package v3.0.1.2^71^ in R v3.4.1^61^. Samples were organized in the heatmap based on UPGMA clustering of Bray–Curtis dissimilarities measured using the “vegdist” function in the “vegan” package v2.4.4^72^.

Gene copy number analyses were performed using scripts from Imamura et al.^9^. Briefly, we calculated median read-depth for each coding region (*c*) in the JPCM5 annotation file and then divided each c value by the median of *c*-values across the chromosome to obtain a normalized copy number estimate (*s*) for each coding region of each sample. We then averaged *s*-values from corresponding coding regions across samples within each of two predefined groups (deletion-carrying and non-deletion type isolates). Coding regions for which group means differed by >0.3 were selected for MWU significance tests using SciPy v1.3.1^73^. Following Bonferroni correction (i.e., dividing the standard *p*-value cutoff of 0.05 by the number of coding regions submitted to MWU), we generated a heatmap of *s*-values at coding regions, which showed significant differences between the two groups, organizing samples by UPGMA clustering of Bray–Curtis similarities as in chromosomal somy visualization above. Coding regions with significant MWU results were also reassessed by ANCOVA using the “car” package v3.0.2^74^ in R v3.4.1^61^ to determine whether *p*-values remained significant after controlling for sample geographic origin. Isolates from Teixeira et al.^33^ (see Supplementary Data 1) were excluded from gene copy number analyses as these had not been made available as complete read-pairs in public sequence archives.

### Monoclonal subcultures and qPCR

Single cell sorting was performed on a MoFLO ASTRIOS Cell Sorter (Beckman Coulter) at the Oswaldo Cruz Institute in Rio de Janeiro, Brazil. *L. infantum* isolates IOCL 2949 and IOCL 3134 entered cell sorting at 10^6^ cells/µl and individual cells were collected in a 96-well plate, each well containing 200 µl Schneider’s medium supplemented with 2% FCS. Wells were inspected five days later using an inverted microscope and liquid from those containing single parasites transferred to separate tubes of NNN. Parasites were pelleted three days later at 1,200 g for 15 min and DNA extracted with DNeasy Blood and Tissue Kit (Qiagen). Primer sequences 5′-ACGATCGGCCTCAAAACACT-3′ (forward) and 5′- GGTGAAGTCTTCGTCCGTGT-3′ (reverse) were designed to target *LinJ.31.2380* (within the chr31 deletion site), and primer sequences 5′-CGAACCTTGGAGCTTCCCTT-3′ (forward) and 5′-TCAAGGTTGTGTCCGTCGAG-3′ (reverse) were designed to target *LinJ.31.2330* (downstream of the chr31 deletion site). IOCL 2666 was used as a reference sample to calibrate the ΔΔCt method described by Livak and Schmittgen^75^. Briefly, qPCR cycle thresholds (Ct values) for both chr31 sequence targets were determined for the samples of interest (IOCL 2949 and 3134, and their monoclonal subcultures) and for IOCL 2666. Ct values for the *LinJ.31.2330* target were assumed to be equivalent between the sample of interest and the reference in the case of equal quantities of input DNA. Deviations from the 1 : 1 ratio for the *LinJ.31.2330* target were used to normalize Ct ratios for the *LinJ.31.2380* target between the sample of interest and the reference. The normalized ratios were considered to represent a fold change estimate of gene dose within the deletion site relative to that within downstream sequence. The qPCR reaction used 0.2 nM primer input and 1× SYBR Green Master Mix with 40 amplification cycles and an annealing temperature of 62 °C. Three experiments were performed per sample, each in technical triplicate. The same fold change estimation protocol was performed in follow-up analysis of monoclonal subcultures 2949 B2 and 2949 G1 using the parental culture IOCL 2949 as the reference.

### Ecto-3’-nucleotidase and ecto-ATPase activity measurement

Ecto-3′-nucleotidase activity was quantified by measuring inorganic phosphate (Pi) release during adenosine 3’-monophosphate (3’-AMP) hydrolysis as in Freitas-Mesquita et al.^28^. Briefly, *L. infantum* promastigotes (10^7^ cells/ml) were incubated at 25 °C for 1 h in 0.5 ml reaction mixture containing 16.0 mM NaCl, 5.4 mM KCl, 5.5 mM d-glucose, 50.0 mM HEPES (pH 7.4), and 3.0 mM 3’-AMP. Reactions were terminated by adding 1.0 ml ice-cold 25% charcoal in 0.1 M HCl and centrifuged at 1500 × *g* for 15 min to remove nonhydrolyzed 3’-AMP. Equal volumes of supernatant and Fiske & Subbarow reagent (0.1 ml each) were mixed to affect the (phosphate-dependent) reduction of ammonium molybdate to phosphomolybdate and absorbance at 660 nm in samples and _Pi_ standards measured after 30 min to derive sample Pi. Ecto-ATPase activity was measured with the same protocol except replacing 3’-AMP with 1.0 mM adenosine 5’-triphosphate and 1.0 mM MgCl_2_. Experiments were performed in technical triplicates using IOCL 2664, 2666, 2972, 3598, and 3634, and monoclonal subcultures 2949 B2 and 3134 B1.

### Statistics and reproducibility

Statistical analyses of the data, sample sizes, number of replicates, and general information on the reproducibility of experiments are depicted at each specific description within the “Methods” section.

### Reporting summary

Further information on research design is available in the Nature Research Reporting Summary linked to this article.

## Supplementary information

Supplementary Information

Description of Additional Supplementary Files

Supplementary Data 1

Supplementary Data 2

Supplementary Data 3

Supplementary Data 4

Supplementary Data 5

Supplementary Data 6

Supplementary Data 7

Supplementary Data 8

Reporting Summary

## Data Availability

New sequence data generated by this study is available at Sequence Read Archive (SRA) BioProject PRJNA658892 (BioSamples SAMN15892565 – SAMN15892623). All other relevant data are available from the corresponding author on reasonable request.

## Code availability

Codes can be accessed at the public repository Zenodo (http://zenodo.org) under the 10.5281/zenodo.42761887^36^.
