# Supplementary material for: Colonization and genetic diversification processes of Leishmania infantum in the Americas
Source: Commun Biol. 2021 Jan 29;4:139. doi: 10.1038/s42003-021-01658-5 (PMC7846609; doi:10.1038/s42003-021-01658-5)
Supplement: Supplementary file 1 — Supplementary Information [file 42003_2021_1658_MOESM1_ESM.pdf]

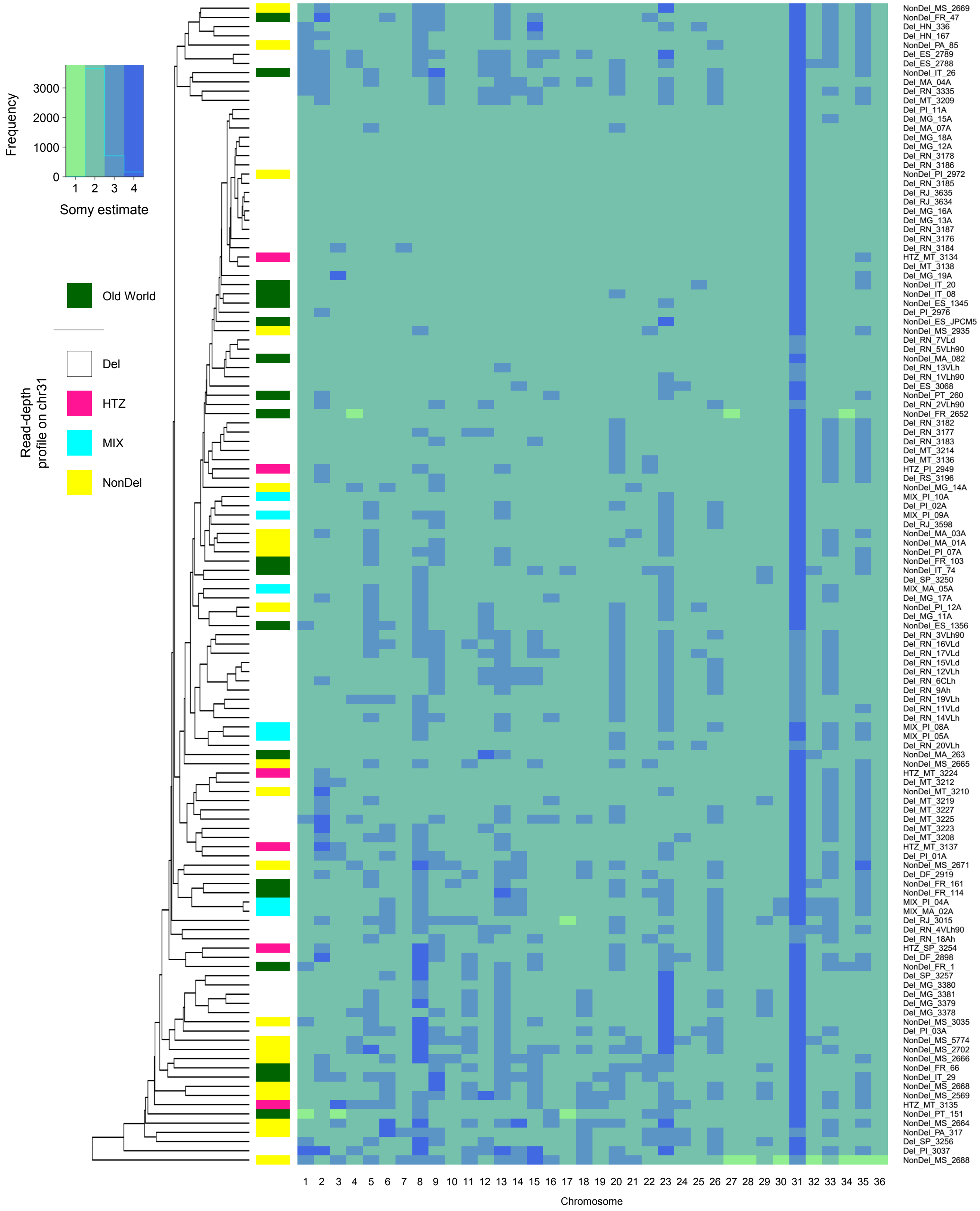

Supplementary Figure 1. Chromosomal copy number variation in New and Old World *L. infantum* isolates. To estimate chromosomal copy numbers for each sample, we calculated mean read-depths for successive 1 kb windows on each chromosome. We then calculated the median of these window means on each chromosome and let the 40th percentile (p40) of the sample's 36 chromosomal medians represent expectations for the disomic state. Somy estimates for each chromosome by median normalization to p40 are plotted in the heatmap. Isolates are ordered on the y-axis by UPGMA clustering of Bray-Curtis dissimilarities. The adjacent column indicates read-depth profiles on (tetrasomic) chr31 according to the color key at left. No correlation to somy is observed.

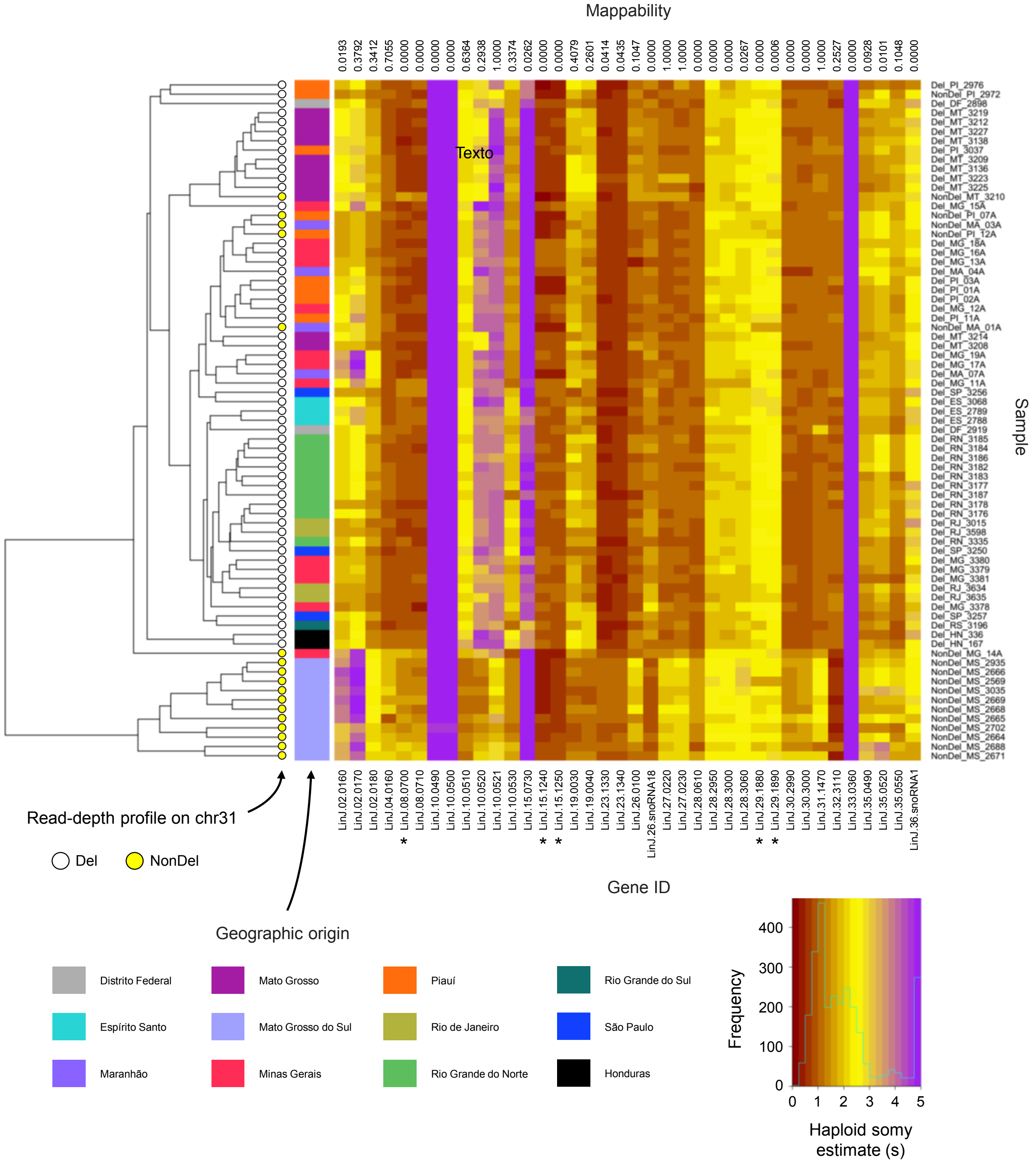

Supplementary Figure 2. Gene copy number variation in Del and NonDel *L. infantum* isolates from the New World. We obtained haploid somy estimates (s) by dividing each coding region's median read-depth (c) by the median of all c values across the chromosome. The heatmap plots s values for all coding regions that differed significantly between Del and NonDel isolates in the New World (see Mann-Whitney U statistics in Supplementary Table 3). Isolates are ordered on the y-axis by UPGMA clustering of Bray-Curtis dissimilarities. Circles at the tips of the tree indicate read-depth profiles on chr31. The adjacent column indicates geographic origin according to the color key below the heatmap. Somy profiles cluster predominantly by geographic origin and only indirectly by chr31 read-depth profile. Only five coding regions differ significantly between Del and NonDel groups after controlling for geographic origin by analysis of covariance (see asterisks). Numbers above the heatmap columns indicate the proportion of uniquely mapping nucleotides within each coding region (see Methods). Poor mappability represents an intrinsic property of genes occurring in multiple paralogs and may explain instances where s > 4 occurs in many samples (i.e., purple columns). Isolates from Teixeira et al. (2017)[35] (see Supplementary Table 1) were excluded from gene copy number analyses because reverse reads were not made available in public sequence archives.

a

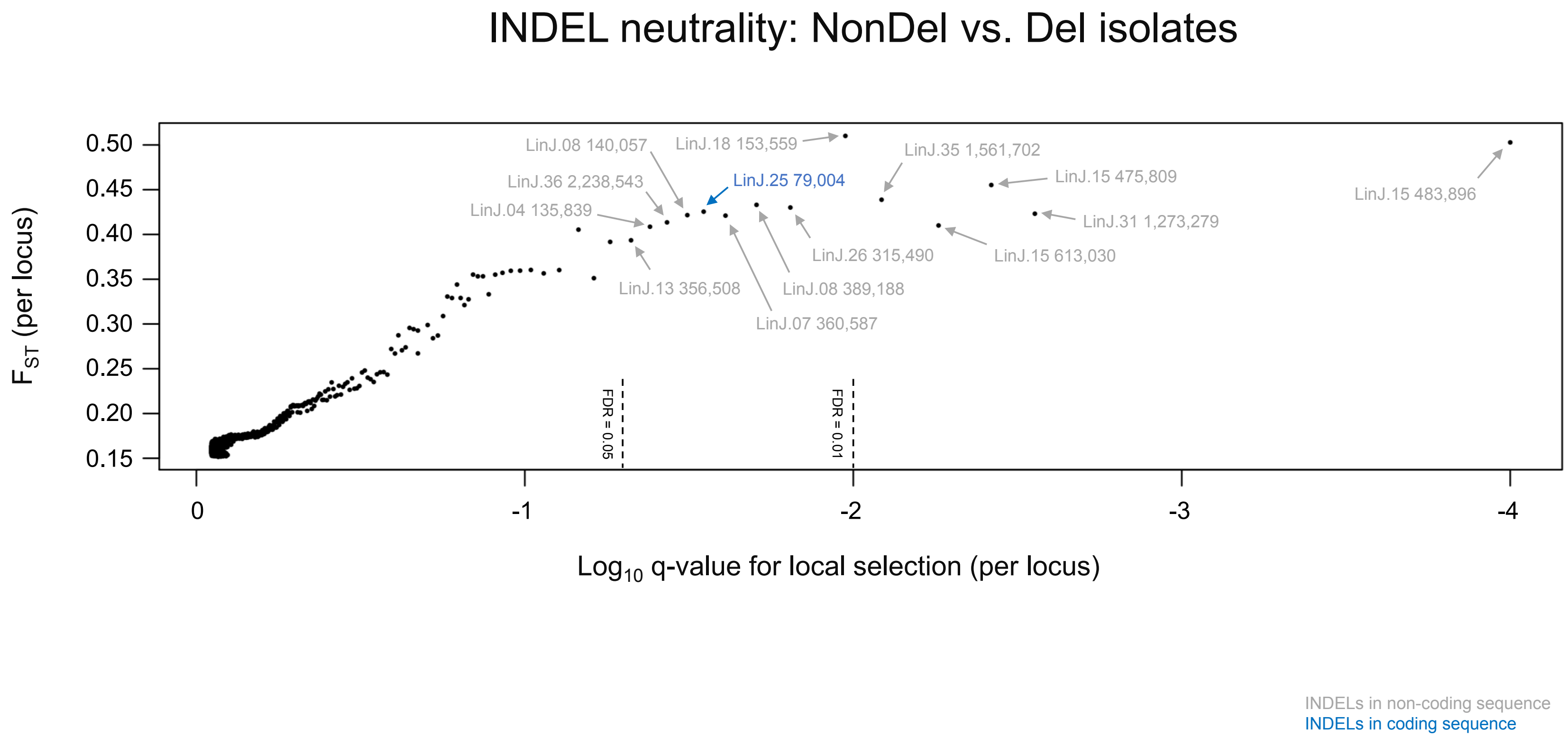

INDEL neutrality: NonDel vs. Del isolates
F_ST (per locus)
0.50
0.45
0.40
0.35
0.30
0.25
0.20
0.15
0
-1
-2
-3
-4
Log10 q-value for local selection (per locus)
FDR = 0.05
FDR = 0.01
LinJ.08 140,057
LinJ.18 153,559
LinJ.36 2,238,543
LinJ.25 79,004
LinJ.04 135,839
LinJ.35 1,561,702
LinJ.15 475,809
LinJ.31 1,273,279
LinJ.15 483,896
LinJ.26 315,490
LinJ.15 613,030
LinJ.13 356,508
LinJ.08 389,188
LinJ.07 360,587
INDELs in non-coding sequence
INDELs in coding sequence

b

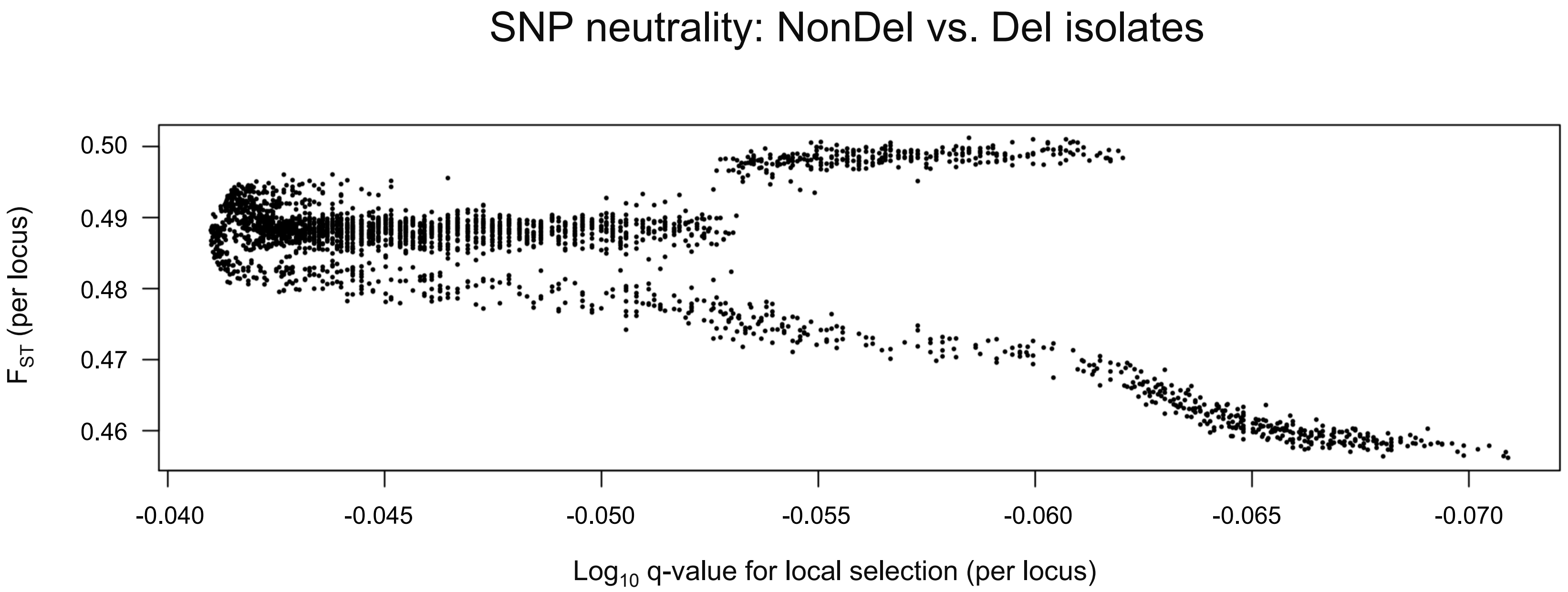

Supplementary Figure 3 Low inter-locus variance in F_{ST} differentiation between Del and NonDel *L. infantum* isolates. **a** F_{ST} values at genome-wide SNP loci range between 0.456 and 0.501. Log_{10} q-values (x-axis) indicate the level of support for selection at each locus. None are significant based on a false discovery rate (FDR) of 5%. SNP sites with genotypes missing in > 50% individuals are excluded from analysis. **b** F_{ST} values at genome-wide INDEL loci range between 0.152 and 0.511. Fourteen outlier loci show significant support for selection at FDR = 5%. Only one of these outliers occurs within coding sequence (blue font) and represents a disruptive inframe deletion in LinJ.25.280. This gene encodes a protein of unknown function on chromosome 25. INDEL sites with genotypes missing in > 50% individuals are excluded from analysis.

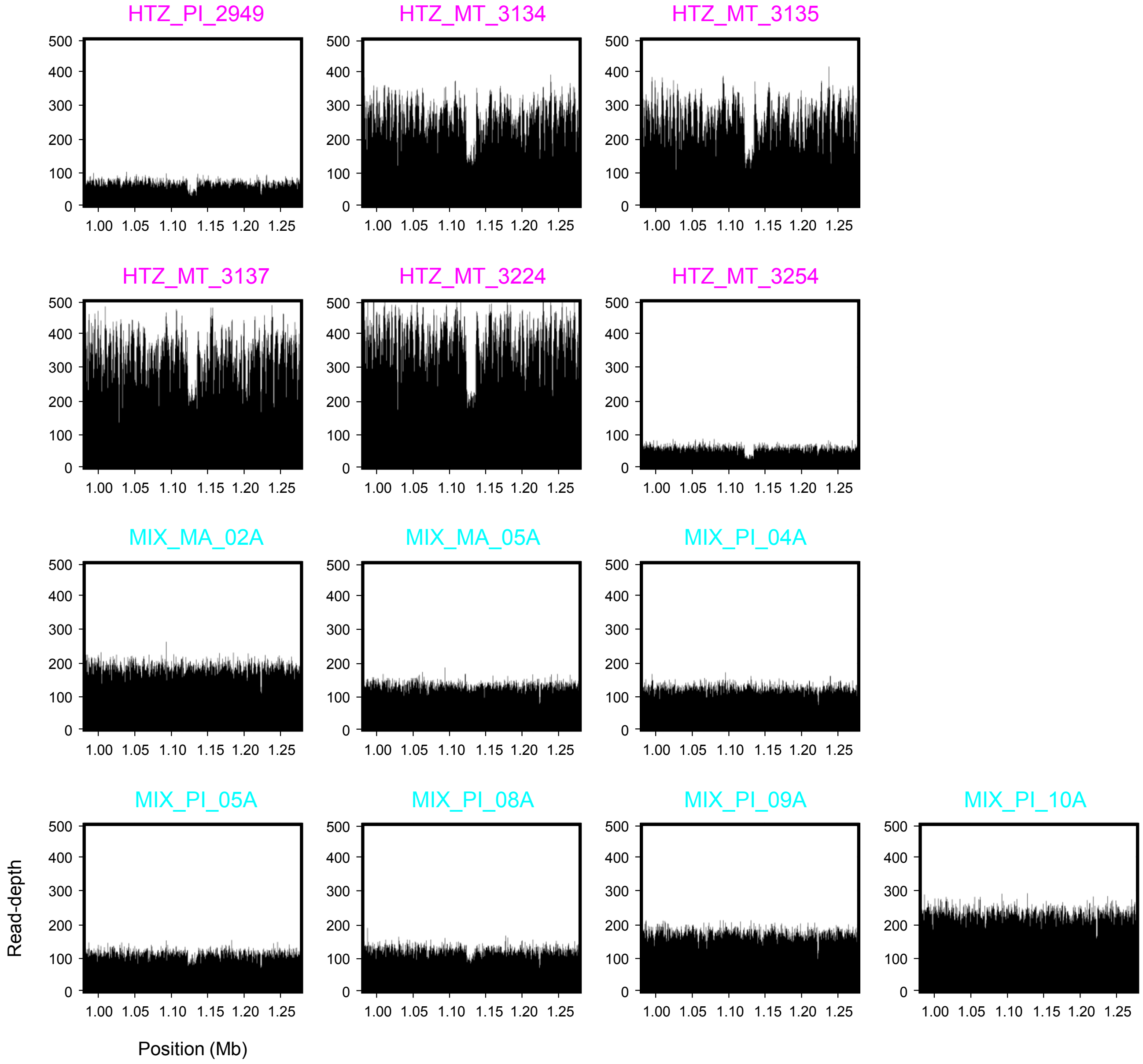

Supplementary Figure 4. Sequence read-depth profiles on chr31 in MIX and HTZ *L. infantum* isolates. Read-depth drops to ca. 50% between 1.122 Mb and 1.135 Mb in all six HTZ isolates. Quantitative PCR confirmed partial deletion at this locus in HTZ cultures derived from single cells. MIX isolates, on the other hand, appear to contain a mixture of NonDel and Del or HTZ profiles based on subclone PCR by Carnielli et al. (2018)[29]. NonDel cells likely predominate in these mixtures given that full read-depth occurs in all but MIX_PI_05A and MIX_PI_08A. Del and HTZ cells may occur more frequently in the latter two isolates.

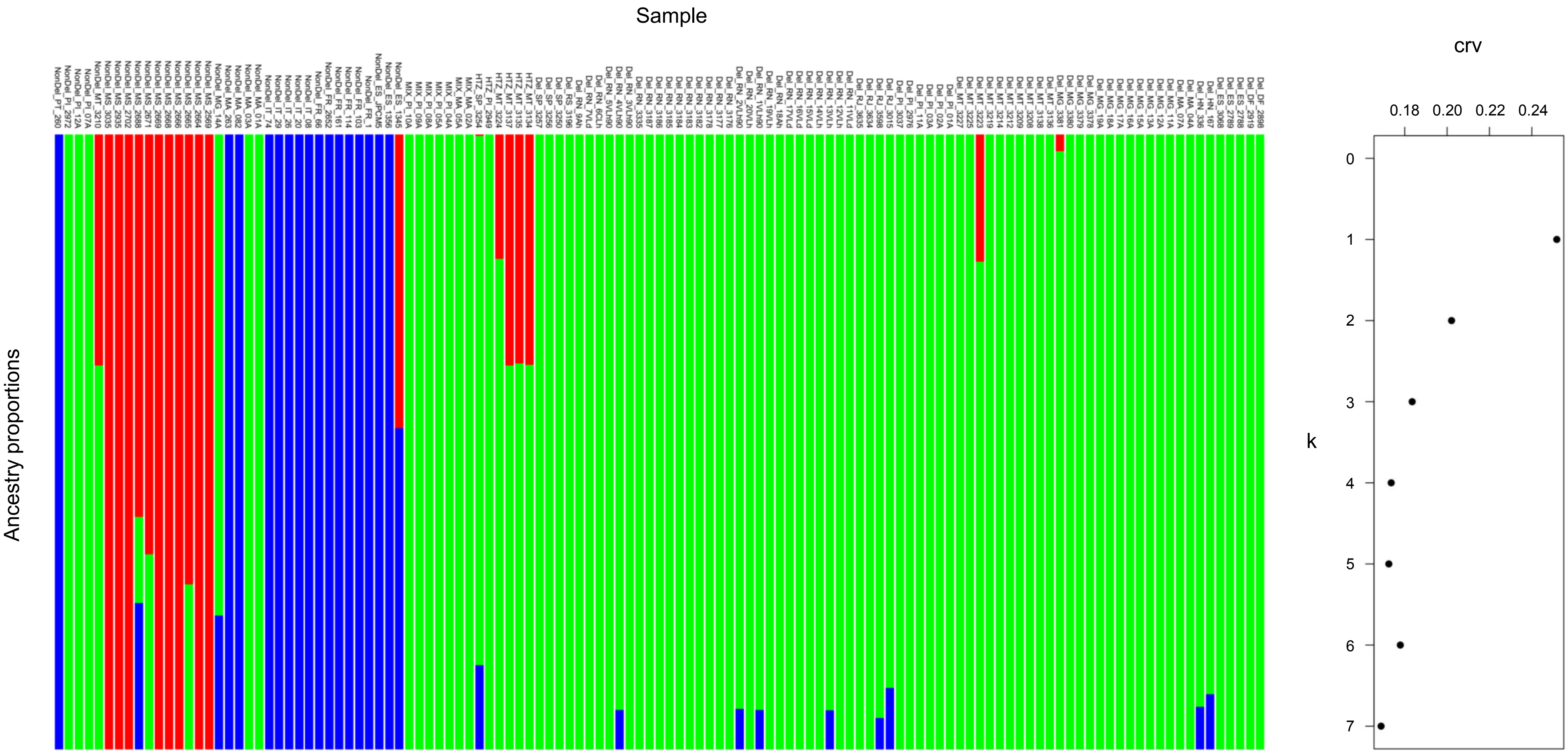

Supplementary Figure 5 Estimated ancestry proportions in New and Old World *L. infantum* isolates. The ADMIXTURE program[45] estimates individual ancestry proportions by optimizing likelihood in the parametric admixture model introduced by Pritchard et al. (2000)[78]. In this model, the number of subpopulations (k) is defined *a priori* and alleles within each individual represent binomial samples from the allele frequencies specific to each subpopulation. Individual ancestry proportions and subpopulation allele frequencies are jointly estimated during model optimization to the genotypes observed. The bar plot summarizes ADMIXTURE results for the *L. infantum* SNP dataset at k = 3. We chose k = 3 based on complementary PCoA analyses (see subsequent figures and text) and because cross-validation error (crv) is low without overfitting the data (right plot). Each column represents one *L. infantum* isolate and relative fill color quantities indicate estimated ancestry proportions. Blue predominates in isolates from the Old World while green and red correspond to New World subpopulations. Several isolates from Mato Grosso and Mato Grosso do Sul show substantial quantities of both red and green, symptomatic of admixture between dissimilar subpopulations in these adjacent states of Brazil. Analyses used all SNP loci for which genotypes are called for all individuals.

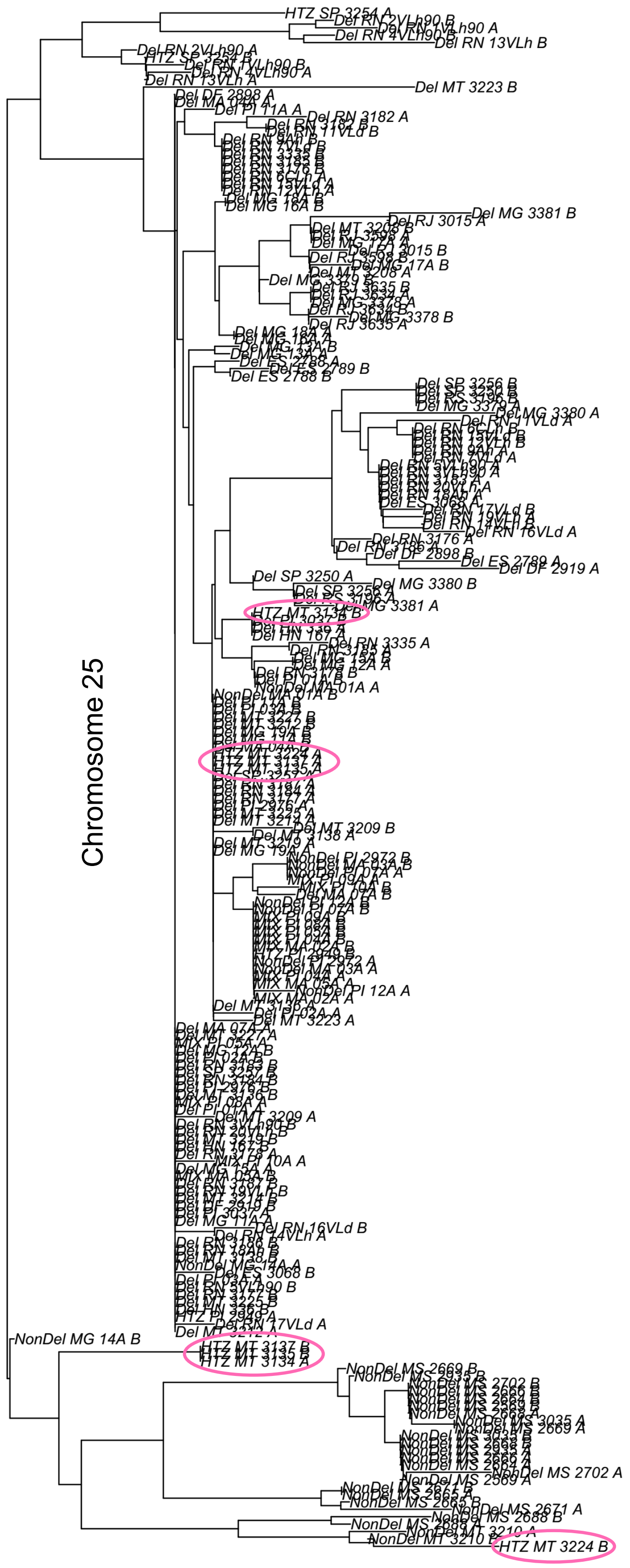
Chromosome 25

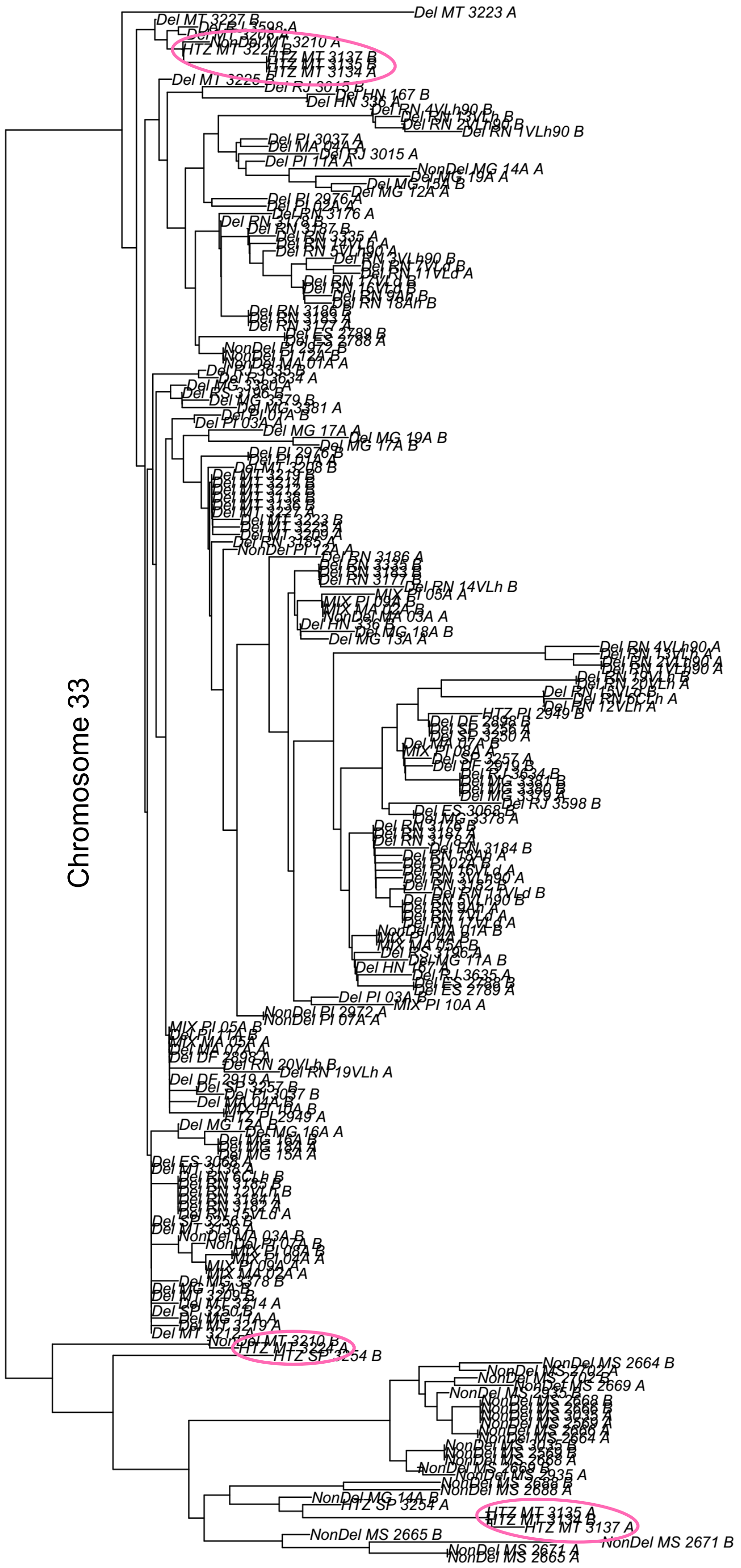

Supplementary Figure 6 Neighbor-joining trees built from phased chromosomes suggest that heterozygous loci in HTZ isolates originate from genetic exchange between divergent haplotypes. Examples are shown from chromosomes 25 and 33. Each tree contains two phased haplotypes per isolate (see label suffixes ‘A’ and ‘B’). Both A and B haplotypes of NonDel isolates from Mato Grosso do Sul (MS) occur within a clade that splits away basally from that containing most other haplotypes. HTZ isolates from the neighboring state of Mato Grosso (MT) often show one haplotype clustering towards this divergent MS clade and the other haplotype showing similarity to Del haplotypes found in MT and other parts of Brazil (see pink circles). A similar trend is observed for isolates such as Del_MT_3223 and NonDel_MT_3210, possibly the result of hybridization with unsampled lineages or secondary hybridizations involving progenitors of this study’s sample set.

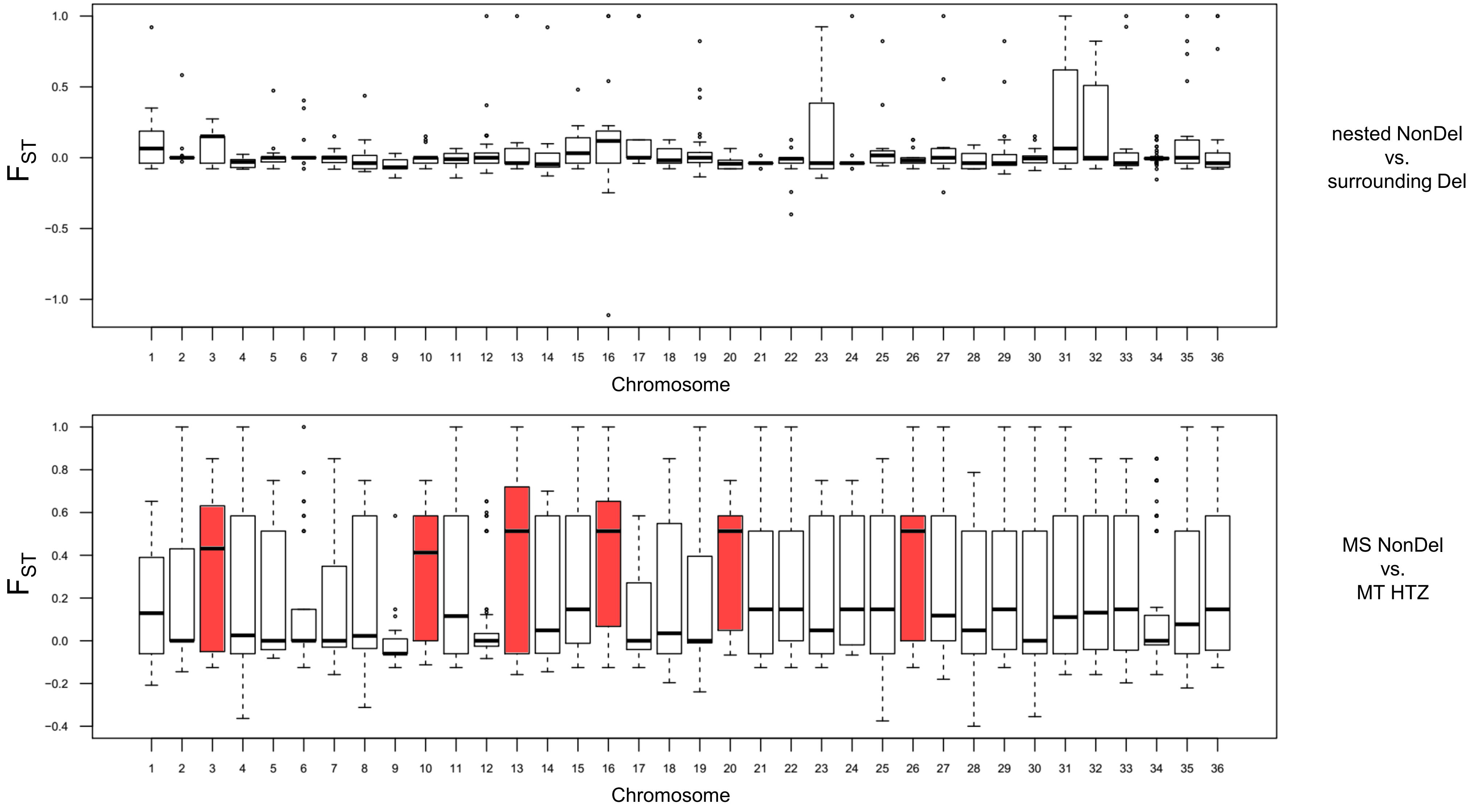

Supplementary Figure 7. NonDel *L. infantum* isolates from Piauí and Maranhão show a subtle increase in divergence to Del isolates on chr31. The top panel shows F_{ST} (Weir and Cockerham) for NonDel_MA_01A, NonDel_MA_03A, NonDel_PI_07A, NonDel_PI_12A and NonDel_PI_2972 relative to Del isolates that surround this nested NonDel group in the phylogenetic tree provided in Fig. 4. These are Del_MA_04A, Del_MA_07A, Del_MG_19A, Del_PI_02A, Del_PI_11A, Del_PI_3037, Del_DF_2898, Del_DF_2919 and Del_SP_3257. Patterns of F_{ST} for putative hybrids (HTZs) from Mato Grosso (MT) relative to NonDel isolates from Mato Grosso do Sul (MS) are distinct. Values are less stable among chromosomes and several medians exceed 0.4 (see red fill).

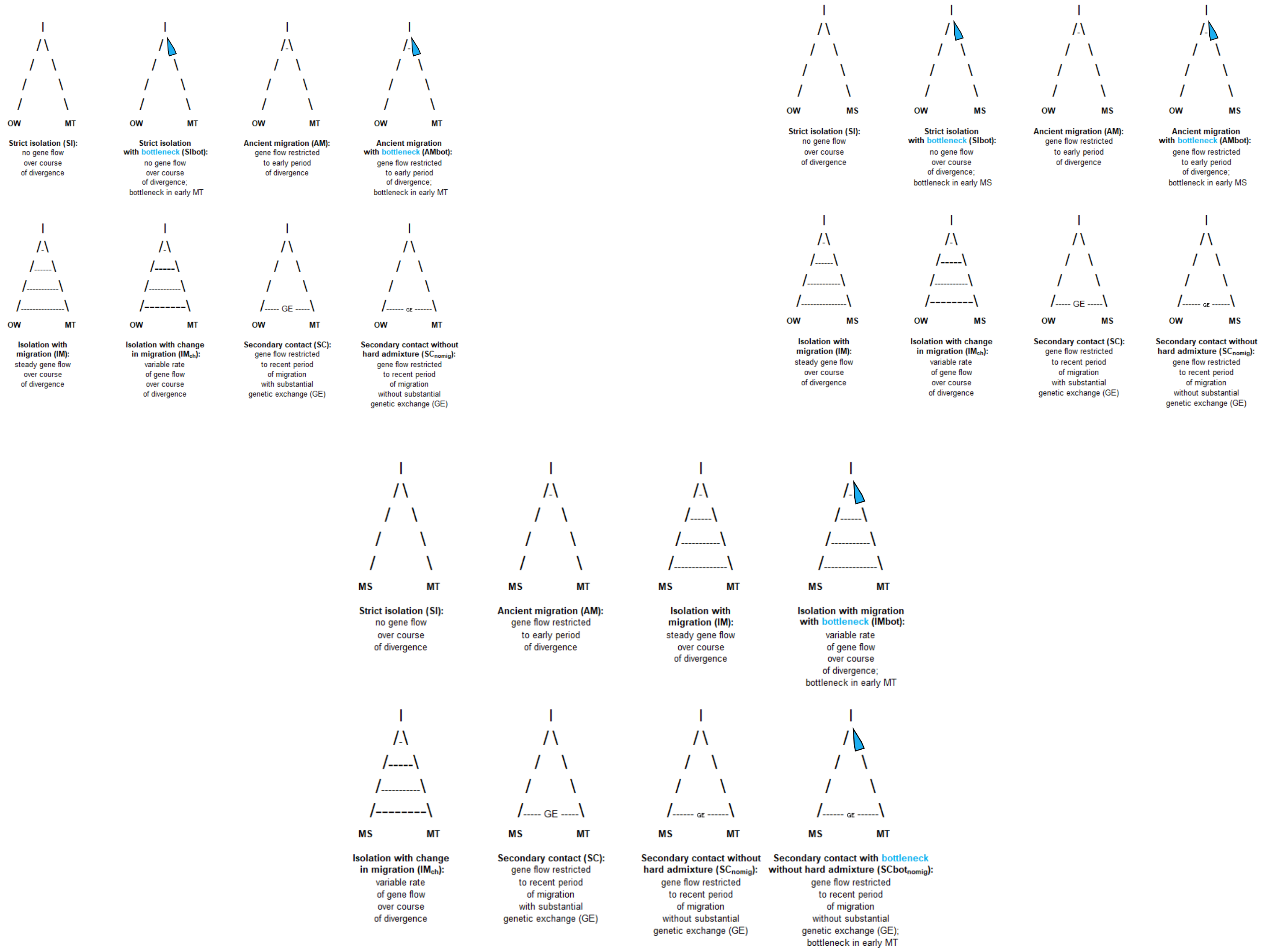

Supplementary Figure 8. Ten scenarios of pairwise divergence simulated using fastsimcoal2[50]. The first set of scenarios depicts divergence between *L. infantum* populations from Mato Grosso (MT) and Mato Grosso do Sul (MS). The second set depicts divergence between populations from MT and the Old World (OW). The third set depicts divergence between populations from MS and OW. Corresponding input syntax is provided in Supplementary Tbl. 7. Dashes horizontal lines indicate gene flow. Blue shapes indicate bottleneck events. Time runs from top (ancestral events) to bottom (present).

Supplementary Note 1

391 sites showed INDELs (small insertions or deletions (up to 30 nt) relative to the JPCM5 reference assembly) in all Del isolates. Of these, however, 260 also showed INDELS in all New World NonDel isolates and 98% occurred in non-coding sequence regions. The two INDELs found in coding regions and present in all Del isolates but not in all New World NonDel isolates (Supplementary Tbl. 4) affect hypothetical proteins LinJ.25.0280 and LinJ.27.0140 without further annotation on TriTrypDB. The INDEL affecting LinJ.25.0280 was the only coding region INDEL suggested to be significantly differentiated between Del and New World NonDel groups based on site-wise F_{ST} differentiation tests (see BayeScan plots in Supplementary Fig. 3). However, complete absence of non-reference genotypes at this locus in Mato Grosso do Sul NonDel isolates suggests that the statistical association reflects common descent as opposed to any direct (selected) response to deletion on chr31.

Forty-one SNPs occurring in coding regions and present in all Del isolates but not in all New World NonDel isolates affected annotated proteins (Supplementary Tbl. 3) but none deviated from neutrality in site-wise F_{ST} differentiation tests (see BayeScan results in Supplementary Fig. 3) and alternative selection analyses including also non-fixed variants (Supplementary Supplementary Tbl. 5).

Supplementary Note 2

A phylogenetic signal on chr31 deletion stop site variation was suggested using different statistical approaches (see Supplementary Code 2). We first ran a Mantel test between stop site differences and genetic distances stratified by read-length differences, yielding $r = 0.35$ and $p = 0.001$. The correlation also remained significant ($r = 0.08$, $p = 0.038$) after excluding samples from Teixeira et al. (2017). These samples generally had lower mapping coverage in repetitive sequence regions, likely because reverse reads were absent from the provided data. In our second approach, we ran a linear model for stop site variation against the explanatory variables read-length and average read-depth. We then applied Blomberg's K to the residuals of this regression and the branch lengths of a neighbor-joining tree. Results suggest a phylogenetic signal on stop site variation, both including ($K = 1.455$, $p = 0.001$) and excluding samples from Teixeira et al. ($K = 1.076$, $p = 0.004$). The signal also remained significant when using median insert size as a third explanatory variable in the latter test ($K = 1.096$, $p = 0.003$). Start site variation did not appear phylogenetically correlated in any of the above analyses.

It is possible that phylogenetically correlated stop site variation reflects independent deletion events among ancestral strains. However, stop sites only differed marginally, ranging between chr31 position 1,135,079 and 1,135,346 (Supplementary Tbl. 2). This range of values implicates the same pair of repetitive sequence blocks (positions 1,122,453 – 1,122,754 and 1,135,162 – 1,135,463) in homologous recombination. If multiple independent deletion events occurred, one might expect different repeat motifs involved in different strains. For example, the repetitive sequence blocks at 1.122 Mb and 1.135 Mb also occur at 1.140 Mb, 1.144 Mb and 1.165 Mb on chr31.

Supplementary Note 3

We verified results of computational phasing for chromosomes 25 and 33 (Supplementary Fig. 6) by assessing HTZ_MT_3135 as a representative of the putative Del/NonDel hybrid genotype found in Mato Grosso state. We concatenated this hybrid representative's computationally phased chromosome 25 haplotype A base identities at all sites for which the sympatric Del representative Del_MT_3136 shows a homozygous (reference or non-reference) genotype. We recorded the fraction of mismatch between HTZ_MT_3135 and Del_MT_3136 at these sites of haplotype A. We then concatenated HTZ_MT_3135's computationally phased chromosome 25 haplotype B base identities at all sites for which the Mato Grosso do Sul NonDel representative NonDel_MS_2671 shows a homozygous (reference or non-reference) genotype. Again, we recorded the fraction of mismatch between the two individuals at these sites of haplotype B. We repeated this procedure for chromosome 33.

Results for chromosome 25:
0 of 9 sites mismatch for haplotype A (HTZ representative vs. Del representative).
4 of 9 sites mismatch for haplotype B (HTZ representative vs. NonDel representative).

Results for chromosome 33:
3 of 21 sites mismatch for haplotype A (HTZ representative vs. Del representative).
6 of 21 sites mismatch for haplotype B (HTZ representative vs. NonDel representative).

These results suggest that computational phasing sufficiently phases one hybrid haplotype to the putative Del progenitor genotype. The second haplotype matches less exactly to the NonDel representative, suggesting that the closest progenitor has not been sampled in this study but is likely related to those samples available from Mato Grosso do Sul. Supplementary Fig. 6's neighbor-joining trees themselves also reflect this reliability in phasing. One HTZ haplotype generally clusters to the same branch tip (no divergence) shared by several Del samples (which are much more homozygous and thus have relatively stable phylogenetic positions for both haplotypes A and B).

Supplementary data deposited at Sequence Read Archive (SRA).

NAME	GEO ORIGIN	SOURCE	SAMPLE	COORD	HOST	R1	R2	COLLECTION DATE	TISSUE ("spleen", "bone marrow", "liver", etc.)	DEPOSITING INSTITUTION (e.g. "Universidade Federal do Mato Grosso do Sul")
MHOM_BR_2006_NMT-HUB402982MO	Brasilia, Brazil	human	Del_DF_2898	15.76 S 47.92 W	Homo sapiens	2898_L006_R1_001.fastq.gz	2898_L006_R2_001.fastq.gz	01-Jan-2006/31-Dec-2006	NA	Universidade de Brasília - UnB
MCAN_BR_2005_NMT-DF544MO	Brasilia, Brazil	dog	Del_DF_2919	15.76 S 47.92 W	Canis familiaris	2919_L006_R1_001.fastq.gz	2919_L006_R2_001.fastq.gz	01-Jan-2005/31-Dec-2005	NA	Universidade de Brasília - UnB
MHOM_BR_2005_DRD	Vitoria, Brazil	human	Del_ES_2788	20.32 S 40.34 W	Homo sapiens	2788_L006_R1_001.fastq.gz	2788_L006_R2_001.fastq.gz	01-Jan-2005/31-Dec-2005	NA	Universidade Federal do Espirito Santo - UFES
MHOM_BR_2005_HRNS-1	Rio Novo do Sul, Brazil	human	Del_ES_2789	20.86 S 40.94 W	Homo sapiens	2789_L006_R1_001.fastq.gz	2789_L006_R2_001.fastq.gz	01-Jan-2005/31-Dec-2005	NA	Universidade Federal do Espirito Santo - UFES
MCAN_BR_2008_CP-18	Pancas, Brazil	dog	Del_ES_3068	19.23 S 40.84 W	Canis familiaris	3068_L006_R1_001.fastq.gz	3068_L006_R2_001.fastq.gz	01-Jan-2008/31-Dec-2008	NA	Universidade Federal do Espirito Santo - UFES
MCAN_BR_2010_CA1	Belo Horizonte, Brazil	dog	Del_MG_3378	19.90 S 43.95 W	Canis familiaris	3378_L006_R1_001.fastq.gz	3378_L006_R2_001.fastq.gz	01-Jan-2010/31-Dec-2010	Bone marrow	Universidade Federal de Minas Gerais - UFMG
MCAN_BR_2010_CA2	Belo Horizonte, Brazil	dog	Del_MG_3379	19.90 S 43.95 W	Canis familiaris	3379_L006_R1_001.fastq.gz	3379_L006_R2_001.fastq.gz	01-Jan-2010/31-Dec-2010	Bone marrow	Universidade Federal de Minas Gerais - UFMG
MCAN_BR_2010_CA3	Belo Horizonte, Brazil	dog	Del_MG_3380	19.90 S 43.95 W	Canis familiaris	3380_L006_R1_001.fastq.gz	3380_L006_R2_001.fastq.gz	01-Jan-2010/31-Dec-2010	Bone marrow	Universidade Federal de Minas Gerais - UFMG
MCAN_BR_2010_CA4	Belo Horizonte, Brazil	dog	Del_MG_3381	19.90 S 43.95 W	Canis familiaris	3381_L006_R1_001.fastq.gz	3381_L006_R2_001.fastq.gz	01-Jan-2010/31-Dec-2010	Bone marrow	Universidade Federal de Minas Gerais - UFMG
MCAN_BR_2009_PATETA	Rondopolis, Brazil	dog	Del_MT_3136	16.49 S 54.62 W	Canis familiaris	3136_S1_L001_R1_001.fastq.gz	3136_S1_L001_R2_001.fastq.gz	01-Jan-2009/31-Dec-2009	Bone marrow	Laboratório de Pesquisa em Leishmaniose
MCAN_BR_2009_BRONCRIS	Rondopolis, Brazil	dog	Del_MT_3138	16.49 S 54.62 W	Canis familiaris	3138_S3_L001_R1_001.fastq.gz	3138_S3_L001_R2_001.fastq.gz	01-Jan-2009/31-Dec-2009	Bone marrow	Laboratório de Pesquisa em Leishmaniose
MCAN_BR_2010_ZEUS	Cuiaba, Brazil	dog	Del_MT_3208	15.61 S 56.06 W	Canis familiaris	3208_S19_L001_R1_001.fastq.gz	3208_S19_L001_R2_001.fastq.gz	01-Jan-2010/31-Dec-2010	Bone marrow	Laboratório de Pesquisa em Leishmaniose
MCAN_BR_2010_ROBI	Cuiaba, Brazil	dog	Del_MT_3209	15.61 S 56.06 W	Canis familiaris	3209_S8_L001_R1_001.fastq.gz	3209_S8_L001_R2_001.fastq.gz	01-Jan-2010/31-Dec-2010	Bone marrow	Laboratório de Pesquisa em Leishmaniose
MCAN_BR_2010_DIMY I	Cuiaba, Brazil	dog	Del_MT_3212	15.61 S 56.06 W	Canis familiaris	3212_S17_L001_R1_001.fastq.gz	3212_S17_L001_R2_001.fastq.gz	01-Jan-2010/31-Dec-2010	Bone marrow	Laboratório de Pesquisa em Leishmaniose
MCAN_BR_2010_MEG	Cuiaba, Brazil	dog	Del_MT_3214	15.61 S 56.06 W	Canis familiaris	3214_S9_L001_R1_001.fastq.gz	3214_S9_L001_R2_001.fastq.gz	01-Jan-2010/31-Dec-2010	Bone marrow	Laboratório de Pesquisa em Leishmaniose
MCAN_BR_2010_TITÃ I	Cuiaba, Brazil	dog	Del_MT_3219	15.61 S 56.06 W	Canis familiaris	3219_S5_L001_R1_001.fastq.gz	3219_S5_L001_R2_001.fastq.gz	01-Jan-2010/31-Dec-2010	Bone marrow	Laboratório de Pesquisa em Leishmaniose
MCAN_BR_2010_BOLINHA III	Cuiaba, Brazil	dog	Del_MT_3223	15.61 S 56.06 W	Canis familiaris	3223_S2_L001_R1_001.fastq.gz	3223_S2_L001_R2_001.fastq.gz	01-Jan-2010/31-Dec-2010	Bone marrow	Laboratório de Pesquisa em Leishmaniose
MCAN_BR_2010_CHITARA I	Cuiaba, Brazil	dog	Del_MT_3225	15.61 S 56.06 W	Canis familiaris	3225_S14_L001_R1_001.fastq.gz	3225_S14_L001_R2_001.fastq.gz	01-Jan-2010/31-Dec-2010	Bone marrow	Laboratório de Pesquisa em Leishmaniose
MCAN_BR_2010_MAGRÃO	Cuiaba, Brazil	dog	Del_MT_3227	15.61 S 56.06 W	Canis familiaris	3227_S15_L001_R1_001.fastq.gz	3227_S15_L001_R2_001.fastq.gz	01-Jan-2010/31-Dec-2010	Bone marrow	Laboratório de Pesquisa em Leishmaniose
MHOM_BR_2006_1406MBS	Teresina, Brazil	human	Del_PI_2976	5.21 S 42.75 W	Homo sapiens	2976_L006_R1_001.fastq.gz	2976_L006_R2_001.fastq.gz	01-Jan-2006/31-Dec-2006	NA	Universidade Federal do Piaui - UFP
MCAN_BR_2007_LIBPI-50	Teresina, Brazil	dog	Del_PI_3037	5.21 S 42.75 W	Canis familiaris	3037_S23_L001_R1_001.fastq.gz	3037_S23_L001_R2_001.fastq.gz	01-Jan-2007/31-Dec-2007	NA	Universidade Federal do Piaui - UFP
MCAN_BR_2015_TUBO9	Barra Mansa, Brazil	dog	Del_RJ_3598	22.55 S 44.16 W	Canis familiaris	MCAN_BR_15_TUBO9_P2_1.fastq.gz	MCAN_BR_25_TUBO9_P2_2.fastq.gz	01-Jan-2015/31-Dec-2015	Spleen	Laboratório de Pesquisa em Leishmaniose
MCAN_BR_2016_90	Barra Mansa, Brazil	dog	Del_RJ_3634	22.55 S 44.16 W	Canis familiaris	MCAN_BR_16_90_P2_1.fastq.gz	MCAN_BR_26_90_P2_2.fastq.gz	01-Jan-2016/31-Dec-2016	Spleen	Laboratório de Pesquisa em Leishmaniose
MCAN_BR_2016_89	Barra Mansa, Brazil	dog	Del_RJ_3635	22.55 S 44.16 W	Canis familiaris	MCAN_BR_16_89_P2_1.fastq.gz	MCAN_BR_26_89_P2_2.fastq.gz	01-Jan-2016/31-Dec-2016	Spleen	Laboratório de Pesquisa em Leishmaniose
MCAN_BR_2010_PV63	Natal, Brazil	dog	Del_RN_3176	5.78 S 35.20 W	Canis familiaris	3176_L006_R1_001.fastq.gz	3176_L006_R2_001.fastq.gz	01-Jan-2010/31-Dec-2010	Spleen	Universidade Federal do Rio Grande do Norte
MCAN_BR_2010_PV64	Natal, Brazil	dog	Del_RN_3177	5.78 S 35.20 W	Canis familiaris	3177_L006_R1_001.fastq.gz	3177_L006_R2_001.fastq.gz	01-Jan-2010/31-Dec-2010	Spleen	Universidade Federal do Rio Grande do Norte
MCAN_BR_2010_PV65	Natal, Brazil	dog	Del_RN_3178	5.78 S 35.20 W	Canis familiaris	3178_L006_R1_001.fastq.gz	3178_L006_R2_001.fastq.gz	01-Jan-2010/31-Dec-2010	Spleen	Universidade Federal do Rio Grande do Norte
MCAN_BR_2010_PV69	Natal, Brazil	dog	Del_RN_3182	5.78 S 35.20 W	Canis familiaris	3182_L006_R1_001.fastq.gz	3182_L006_R2_001.fastq.gz	01-Jan-2010/31-Dec-2010	Spleen	Universidade Federal do Rio Grande do Norte
MCAN_BR_2010_PV71	Natal, Brazil	dog	Del_RN_3183	5.78 S 35.20 W	Canis familiaris	3183_L006_R1_001.fastq.gz	3183_L006_R2_001.fastq.gz	01-Jan-2010/31-Dec-2010	Spleen	Universidade Federal do Rio Grande do Norte
MCAN_BR_2010_PV72	Natal, Brazil	dog	Del_RN_3184	5.78 S 35.20 W	Canis familiaris	3184_L006_R1_001.fastq.gz	3184_L006_R2_001.fastq.gz	01-Jan-2010/31-Dec-2010	Spleen	Universidade Federal do Rio Grande do Norte
MCAN_BR_2010_PV73	Natal, Brazil	dog	Del_RN_3185	5.78 S 35.20 W	Canis familiaris	3185_L006_R1_001.fastq.gz	3185_L006_R2_001.fastq.gz	01-Jan-2010/31-Dec-2010	Spleen	Universidade Federal do Rio Grande do Norte
MCAN_BR_2010_PV74	Natal, Brazil	dog	Del_RN_3186	5.78 S 35.20 W	Canis familiaris	3186_L006_R1_001.fastq.gz	3186_L006_R2_001.fastq.gz	01-Jan-2010/31-Dec-2010	Spleen	Universidade Federal do Rio Grande do Norte
MCAN_BR_2010_PV75	Natal, Brazil	dog	Del_RN_3187	5.78 S 35.20 W	Canis familiaris	3187_L006_R1_001.fastq.gz	3187_L006_R2_001.fastq.gz	01-Jan-2010/31-Dec-2010	Spleen	Universidade Federal do Rio Grande do Norte
MCAN_BR_2011_PV 128	Natal, Brazil	dog	Del_RN_3335	5.78 S 35.20 W	Canis familiaris	3335_L006_R1_001.fastq.gz	3335_L006_R2_001.fastq.gz	01-Jan-2011/31-Dec-2011	Spleen	Universidade Federal do Rio Grande do Norte
MCAN_BR_2010_LUNA II	Uruguaiana, Brazil	dog	Del_RS_3196	29.77 S 57.07 W	Canis familiaris	3196_L008_R1_001.fastq.gz	3196_L008_R2_001.fastq.gz	01-Jan-2010/31-Dec-2010	Bone marrow	Centro Estadual de Vigilância em Saúde / SES-RS
MCAN_BR_2009_CLV9	Embu das Artes, Brazil	dog	Del_SP_3250	23.65 S 46.85 W	Canis familiaris	3250_L006_R1_001.fastq.gz	3250_L006_R2_001.fastq.gz	01-Jan-2009/31-Dec-2009	Spleen	Universidade de São Paulo - USP
MCAN_BR_2009_CLV22	Embu das Artes, Brazil	dog	Del_SP_3256	23.65 S 46.85 W	Canis familiaris	3256_L006_R1_001.fastq.gz	3256_L006_R2_001.fastq.gz	01-Jan-2009/31-Dec-2009	Linfonode	Universidade de São Paulo - USP
MCAN_BR_2011_IMTS-14	Embu das Artes, Brazil	dog	Del_SP_3257	23.65 S 46.85 W	Canis familiaris	3257_L006_R1_001.fastq.gz	3257_L006_R2_001.fastq.gz	01-Jan-2011/31-Dec-2011	NA	Universidade de São Paulo - USP
MCAN_BR_2009_GRANDÃO I	Rondopolis, Brazil	dog	HTZ_MT_3134	16.49 S 54.62 W	Canis familiaris	3134_S6_L001_R1_001.fastq.gz	3134_S6_L001_R2_001.fastq.gz	01-Jan-2009/31-Dec-2009	Liver	Laboratório de Pesquisa em Leishmaniose
MCAN_BR_2009_GRANDÃO II	Rondopolis, Brazil	dog	HTZ_MT_3135	16.49 S 54.62 W	Canis familiaris	3135_S11_L001_R1_001.fastq.gz	3135_S11_L001_R2_001.fastq.gz	01-Jan-2009/31-Dec-2009	Liver	Laboratório de Pesquisa em Leishmaniose
MCAN_BR_2009_SOL	Rondopolis, Brazil	dog	HTZ_MT_3137	16.49 S 54.62 W	Canis familiaris	3137_S13_L001_R1_001.fastq.gz	3137_S13_L001_R2_001.fastq.gz	01-Jan-2009/31-Dec-2009	Spleen	Laboratório de Pesquisa em Leishmaniose
MCAN_BR_2010_GUG	Cuiaba, Brazil	dog	HTZ_MT_3224	15.61 S 56.06 W	Canis familiaris	3224_S24_L001_R1_001.fastq.gz	3224_S24_L001_R2_001.fastq.gz	01-Jan-2010/31-Dec-2010	Spleen	Laboratório de Pesquisa em Leishmaniose
MCAN_BR_2004_LIBPI-18	Teresina, Brazil	dog	HTZ_PI_2949	5.21 S 42.75 W	Canis familiaris	2949_L006_R1_001.fastq.gz	2949_L006_R2_001.fastq.gz	01-Jan-2004/31-Dec-2004	NA	Universidade Federal do Piaui - UFP
MCAN_BR_2009_CLV17	Embu das Artes, Brazil	dog	HTZ_SP_3254	23.65 S 46.85 W	Canis familiaris	3254_L006_R1_001.fastq.gz	3254_L006_R2_001.fastq.gz	01-Jan-2009/31-Dec-2009	Spleen	Universidade de São Paulo - USP
MCAN_BR_2003_HUGER	Campo Grande, Brazil	dog	NonDel_MS_2569	20.48 S 54.60 W	Canis familiaris	2569_S7_L001_R1_001.fastq.gz	2569_S7_L001_R2_001.fastq.gz	01-Jan-2003/31-Dec-2003	NA	Universidade Federal do Mato Grosso do Sul - UFMS
MCAN_BR_2002_LVV-135	Campo Grande, Brazil	dog	NonDel_MS_2664	20.48 S 54.60 W	Canis familiaris	2664_L006_R1_001.fastq.gz	2664_L006_R2_001.fastq.gz	01-Jan-2002/31-Dec-2002	NA	Universidade Federal do Mato Grosso do Sul - UFMS
MCAN_BR_2002_LVV-136	Campo Grande, Brazil	dog	NonDel_MS_2665	20.48 S 54.60 W	Canis familiaris	2665_S16_L001_R1_001.fastq.gz	2665_S16_L001_R2_001.fastq.gz	01-Jan-2002/31-Dec-2002	NA	Universidade Federal do Mato Grosso do Sul - UFMS
MCAN_BR_2002_LVV-137	Tres Lagoas, Brazil	dog	NonDel_MS_2666	20.73 S 51.68 W	Canis familiaris	2666_S12_L001_R1_001.fastq.gz	2666_S12_L001_R2_001.fastq.gz	01-Jan-2002/31-Dec-2002	NA	Universidade Federal do Mato Grosso do Sul - UFMS
MCAN_BR_2002_LVV-139	Tres Lagoas, Brazil	dog	NonDel_MS_2668	20.73 S 51.68 W	Canis familiaris	2668_S10_L001_R1_001.fastq.gz	2668_S10_L001_R2_001.fastq.gz	01-Jan-2002/31-Dec-2002	NA	Universidade Federal do Mato Grosso do Sul - UFMS
MCAN_BR_2002_LVV-140	Tres Lagoas, Brazil	dog	NonDel_MS_2669	20.73 S 51.68 W	Canis familiaris	2669_S21_L001_R1_001.fastq.gz	2669_S21_L001_R2_001.fastq.gz	01-Jan-2002/31-Dec-2002	NA	Universidade Federal do Mato Grosso do Sul - UFMS
MCAN_BR_2002_LVV-145	Tres Lagoas, Brazil	dog	NonDel_MS_2671	20.73 S 51.68 W	Canis familiaris	2671_L006_R1_001.fastq.gz	2671_L006_R2_001.fastq.gz	01-Jan-2002/31-Dec-2002	NA	Universidade Federal do Mato Grosso do Sul - UFMS
MHOM_BR_2003_phufms-155	Tres Lagoas, Brazil	human	NonDel_MS_2702	20.73 S 51.68 W	Homo sapiens	2702_L006_R1_001.fastq.gz	2702_L006_R2_001.fastq.gz	01-Jan-2003/31-Dec-2003	NA	Universidade Federal do Mato Grosso do Sul - UFMS
MCAN_BR_2007_CG-2	Campo Grande, Brazil	human	NonDel_MS_2935	20.48 S 54.60 W	Homo sapiens	2935_S20_L001_R1_001.fastq.gz	2935_S20_L001_R2_001.fastq.gz	01-Jan-2007/31-Dec-2007	NA	Universidade Federal do Mato Grosso do Sul - UFMS
MCAN_BR_2010_CALSITO I	Campo Grande, Brazil	dog	NonDel_MS_3035	20.48 S 54.60 W	Canis familiaris	3035_S18_L001_R1_001.fastq.gz	3035_S18_L001_R2_001.fastq.gz	01-Jan-2007/31-Dec-2007	NA	Universidade Federal do Mato Grosso do Sul - UFMS
MHOM_BR_2005_742EMS	Cuiaba, Brazil	dog	NonDel_MT_3210	15.61 S 56.06 W	Canis familiaris	3210_S4_L001_R1_001.fastq.gz	3210_S4_L001_R2_001.fastq.gz	01-Jan-2010/31-Dec-2010	Spleen	Laboratório de Pesquisa em Leishmaniose
LLM1345p2	Teresina, Brazil	human	NonDel_PI_2972	5.21 S 42.75 W	Homo sapiens	2972_L006_R1_001.fastq.gz	2972_L006_R2_001.fastq.gz	01-Jan-2005/31-Dec-2005	NA	Universidade Federal do Piaui - UFP
LLM1356p2	Madrid, Spain	dog	NonDel_ES_1345	40.41 N 3.69 W	Canis familiaris	LLM1345_1.fastq.gz	LLM1345_2.fastq.gz	01-Jan-2004/31-Dec-2004	Bone marrow	Instituto de Salud Carlos III, WHO Collaborating Center for Leishmaniasis/ Spain
MCAN/ES/98/LLM-724	Madrid, Spain	dog	NonDel_ES_1356	40.41 N 3.69 W	Canis familiaris	LLM1356_1.fastq.gz	LLM1356_2.fastq.gz	01-Jan-2004/31-Dec-2004	Bone marrow	Instituto de Salud Carlos III, WHO Collaborating Center for Leishmaniasis/ Spain
MHOM_BR_2007_ARL	Madrid, Spain	dog	NonDel_ES_JPCM5	40.41 N 3.69 W	Canis familiaris	LRef_S25_L001_R1_001.fastq.gz	LRef_S25_L001_R2_001.fastq.gz	01-Jan-1998/31-Dec-1998	Bone marrow	Instituto de Salud Carlos III, WHO Collaborating Center for Leishmaniasis/ Spain
