# Supplementary material for: Colonization and genetic diversification processes of Leishmania infantum in the Americas
Source: Commun Biol. 2021 Jan 29;4:139. doi: 10.1038/s42003-021-01658-5 (PMC7846609; doi:10.1038/s42003-021-01658-5)
Supplement: Supplementary file 2 — Description of Additional Supplementary Files [file 42003_2021_1658_MOESM2_ESM.pdf]

Description of Additional Supplementary Files

File name: **Supplementary data 1.** *L. infantum* isolates analyzed by whole-genome sequencing or quantitative polymerase chain reaction. Abbreviations: IOCL (Coleção de Leishmania do Instituto Oswaldo Cruz); NRD (average nuclear read-depth); PCR (polymerase chain reaction testing for presence/absence of the chr31 deletion locus); WGS (whole-genome sequencing); NA (not applicable); ND (not determined); WSI (Wellcome Sanger Institute – unpublished sequencing data kindly provided by the parasite genomics group).

File name: **Supplementary data 2.** Boundaries of the > 12 kb deletion on chr31. Start and stop sites (listed in ascending order) are defined as the most downstream and upstream nucleotide positions (respectively) with zero mapping depth between .bam file positions 1,122,000 and 1,136,000. Contiguous upstream and downstream positions of these start and stop sites (respectively) also have zero mapping depth.

File name: **Supplementary data 3.** Gene copy number variation between Del and NonDel *L. infantum* isolates of the New World. Haploid somy estimates (s) in 89 coding regions differed by more than 0.3 between Del and NonDel groups. This table describes results from the 42 of these 90 regions that appear statistically significant in Mann-Whitney U (MWU) analysis using a Bonferroni-corrected p-value cut-off of 0.05 / 89 = 0.000562. A Bray-Curtis distance matrix calculated from the s values of these significantly differentiated regions was used to cluster samples in a heatmap in Supplementary Fig. 2. The heatmap excludes values for the four genes within the chr31 deletion locus (grey font) in order to assess whether other copy number changes correlate with this trait. The heatmap exposes a strong correlation between geographic origin and s. We therefore reassessed all 42 regions by analysis of covariance (ANCOVA) with geographic origin applied as a covariate to Del vs. NonDel chr31 read-depth profile. Only nine coding regions remain significant (bold font) with the additional covariate applied. The last column of this table also indicates the proportion of uniquely mapping nucleotides within each coding region (see Methods). Poor mappability is likely for genes occurring in multiple paralogs and may explain instances where s > 4 in Supplementary Fig. 2. Product descriptions were obtained from the JPCM5 annotation file available at https://tritrypdb.org/common/downloads/release-24/LinfantumJPCM5/gff/data/. Additional abbreviations: n (sample size); Δs (mean s in Del minus mean s in NonDel isolates).

File name: **Supplementary data 4.** Short insertion-deletion and single-nucleotide variants present in all Del isolates but not in all NonDel *L. infantum* isolates of the New World. Variant effect and impact was determined by SNPEff using the JPCM5 annotation file available at https://tritrypdb.org/common/downloads/release-33/LinfantumJPCM5/gff/data/. Abbreviations: chr. (chromosome); pos. (position); single-nucleotide polymorphism (SNP); insertion-deletion variant (INDEL).

File name: **Supplementary data 5.** Short insertion-deletion and single-nucleotide variants prevalent (> 70%) in Del but uncommon (< 50%) in NonDel isolates of the New World. Variant effect and impact was determined by SNPEff using the JPCM5 annotation file available at https://tritrypdb.org/common/downloads/release-33/LinfantumJPCM5/gff/data/. Abbreviations: chr. (chromosome); pos. (position); single-nucleotide polymorphism (SNP); insertion-deletion variant (INDEL).

File name: **Supplementary data 6.** Significant heterozygosity increases in HTZ *L. infantum* groups. The Kruskall-Wallis rank sum test indicates that genome-wide inbreeding coefficients (FIS values) differ among Del, HTZ, MIX and New World (NW) NonDel groups (p-value < 0.001). This table lists FIS medians and p-values from post-hoc pairwise comparisons using the Tukey and Kramer (Nemenyi) test. Results indicate significant FIS reductions in HTZ groups. Hyphens replace redundant comparisons. Medians for raw counts of heterozygous loci (Het.) are also shown. Het. values produce analogous values in Kruskal-Wallis and Nemenyi tests (not shown).

File name: **Supplementary Data 7**. Source Data to Figure 2. Original table containing source data to the referred analysis.

File name: **Supplementary Data 8.** Source Data to Figure 6. Original table containing source data to the referred analysis.
