# Supplementary material for: Colonization and genetic diversification processes of Leishmania infantum in the Americas
Source: Commun Biol. 2021 Jan 29;4:139. doi: 10.1038/s42003-021-01658-5 (PMC7846609; doi:10.1038/s42003-021-01658-5)
Supplement: Supplementary file 11 — Reporting Summary [file 42003_2021_1658_MOESM11_ESM.pdf]

nature research

Corresponding author(s): Mariana BOITÉ

Last updated by author(s): Dec 6, 2020

Reporting Summary

Nature Research wishes to improve the reproducibility of the work that we publish. This form provides structure for consistency and transparency in reporting. For further information on Nature Research policies, see our Editorial Policies and the Editorial Policy Checklist.

Statistics

For all statistical analyses, confirm that the following items are present in the figure legend, table legend, main text, or Methods section.

n/a	Confirmed	
☐	☒	The exact sample size (*n*) for each experimental group/condition, given as a discrete number and unit of measurement
☐	☒	A statement on whether measurements were taken from distinct samples or whether the same sample was measured repeatedly
☐	☒	The statistical test(s) used AND whether they are one- or two-sided<br>*Only common tests should be described solely by name; describe more complex techniques in the Methods section.*
☐	☒	A description of all covariates tested
☐	☒	A description of any assumptions or corrections, such as tests of normality and adjustment for multiple comparisons
☐	☒	A full description of the statistical parameters including central tendency (e.g. means) or other basic estimates (e.g. regression coefficient) AND variation (e.g. standard deviation) or associated estimates of uncertainty (e.g. confidence intervals)
☐	☒	For null hypothesis testing, the test statistic (e.g. *F*, *t*, *r*) with confidence intervals, effect sizes, degrees of freedom and *P* value noted<br>*Give P values as exact values whenever suitable.*
☐	☒	For Bayesian analysis, information on the choice of priors and Markov chain Monte Carlo settings
☒	☐	For hierarchical and complex designs, identification of the appropriate level for tests and full reporting of outcomes
☒	☐	Estimates of effect sizes (e.g. Cohen's *d*, Pearson's *r*), indicating how they were calculated

Our web collection on statistics for biologists contains articles on many of the points above.

Software and code

Policy information about availability of computer code

Data collection	N/a
Data analysis	BWA-mem v0.7.3 (suffix tree-based sequence read alignment); Genome Analysis Toolkit v3.7.0 (genetic variant discovery and genotyping); BEAGLE v4.1 (haplotype phasing by use of localized haplotype clustering); TreeMix v1.13 (phylogenetic tree-to-graph optimization); fastsimcoal2 v2.5.2 (coalescent simulation of genomic diversity); ARLSUMSTAT v3.5.2 (population genetic analyses); BayeScan v2.1 (selection analysis); SNPEff v.3t (variant annotation); Genomic Multi-tool v1.376 (indexing and querying genomic data files); SAMtools v0.1.18 (sequence read alignment processing); SciPy v.1.3.1 (mathematical tools in python); IQ-Tree v1.5.4 (phylogenetic tree construction); ADMIXTURE v1.3 (ancestry inference); Picard v1.85 (high-throughput sequence data handling); VCFtools v0.1.13 (variant-call-format data handling); Packages used in R v3.4.1: stats v3.4.1 (statistical tools); adegenet v2.1.1 (multivariate analysis of genetic markers); ape v5.0 (phylogenetic analyses); abcrf v1.7 (approximate Bayesian computation via random forests); gplots v3.0.1.2 (data plotting); vegan v2.4.4 (descriptive community analysis); car v3.0.2 (applied regression); Genome Analysis Toolkit (GATK) v3.7.0; Genomic Multi-tool; Samtools view (version 1.3) and BEDTools genomecov (version 2.25.0); Graphpad Software V5.

For manuscripts utilizing custom algorithms or software that are central to the research but not yet described in published literature, software must be made available to editors and reviewers. We strongly encourage code deposition in a community repository (e.g. GitHub). See the Nature Research guidelines for submitting code & software for further information.

Data

Policy information about availability of data

All manuscripts must include a data availability statement. This statement should provide the following information, where applicable:

- Accession codes, unique identifiers, or web links for publicly available datasets
- A list of figures that have associated raw data
- A description of any restrictions on data availability

New sequence data generated by this study is available at Sequence Read Archive (SRA) BioProject PRJNA658892 (BioSamples SAMN15892565 – SAMN15892623). All other relevant data are available from the corresponding author on reasonable request.

Field-specific reporting

Please select the one below that is the best fit for your research. If you are not sure, read the appropriate sections before making your selection.

☐ Life sciences ☐ Behavioural & social sciences ☒ Ecological, evolutionary & environmental sciences

For a reference copy of the document with all sections, see nature.com/documents/nr-reporting-summary-flat.pdf

Ecological, evolutionary & environmental sciences study design

All studies must disclose on these points even when the disclosure is negative.

Study description	The study explores signals of the Leishmania infantum parasite genomic adaptation to the New World and assess the biological consequences. Leishmania infantum is an ancient Old World parasite that has expanded across much of Latin America within the last five hundred years. Its rapid ecological adaptation represents a case study from which to understand parasite molecular mechanisms that facilitate range expansion. We reconstructed demographic histories based on whole-genome analysis of New (n=107) and Old World (n=19) L. infantum genomes, tracing how post-introduction bottleneck and migration events have contributed to parasite population genetic structure. A comparative genomic analysis of these 126 New World and Old World L. infantum genomes was employed against the JPCM5 reference assembly. We demonstrate loss of ecto-3'-nucleotidase function, an enzyme coded within the deleted locus, (n=12; experimental triplicates) coupled to increased ectoATPase activity in deletion-carrying strains (n=4). We further demonstrate altered phenotypes in heterozygous deletion mutants that are the product of hybridization events between deletion-carrying and non-deletion strains. The "heterozygosity" of the deleted site was characterized by qPCR of monoclonal cultured parasites (n=11) in technical and experimental triplicates.
Research sample	Leishmania infantum, L. donovani and L. archibaldi are species from the L. donovani complex, part of the Leishmania (Leishmania) subgenus. L. infantum - the targeted species in our study - was imported to the Americas within the last five hundred years during the colonization process. Nowadays the parasite is the main agent of visceral leishmaniasis and has successfully expanded across the continent. Aiming to explore this process by genomic/demographic analysis we stablished a sampling strategy to geographically cover such importation process and distribution in the New World, mainly in Brazil (responsible for around 90% of the visceral cases in the Americas). Therefore, we select samples from different Brazilian regions and bordering countries. specially from important endemic Brazilian regions in which complex transmission cycles are described. Most of the samples were available at the biobank CLIOC (Coleção de Leishmania da Fundação Oswaldo Cruz) but we also included available genomes at SRA in most of the analysis to complement the demographic / phylogenomic history.
Sampling strategy	Most of the New World Leishmania infantum strains (n=133) were obtained from a biobank (Coleção de Leishmania do Instituto Oswaldo Cruz - CLIOC - WDCM 731 - FIOCRUZ. Brazil http://clioc.fiocruz.br) to be further subjected to either WG-NGS (n=58), qPCR (n=75) and/or enzymatic phenotypic assay (n=7). Criteria for selection included geographic origin and availability. All Brazilian Major Geographic Regions were represented in a relative frequency, which is dependent of how regular public/researchers health professional from each area successfully isolate and ship the samples to the biobank. The study did not involve field work or fresh sample collection. The well-known Brazilian endemic regions were properly represented. Until now, our sampling strategy achieved the most robust and geographically representative number of American L. infantum strains assessed by a phylogenomic/ demographic study. Conditions did not allow a prior sample-size calculation, though. Additionally, most of the online available L. infantum genomes were selected. From online repositories we assessed genomes from Morocco (n=2), Portugal (n=2), France (n=7), Italy (n= 5), Spain (n=3), Panama (n=2) and Honduras (n=2).<br>For phenotypic assays the distinct deleted, non-deleted and heterozygous samples (at least 2 of each genotype) represented biological replicates for the targeted genomic trait.
Data collection	n/a
Timing and spatial scale	Assessing repository-available genomes and performing WGS: September 2017 to September 2019; Obtaining samples from the biobank for qPCR data collection: March 2018 to September 2018; Enzymatic assays: May 2018 to September 2018.
Data exclusions	All samples used in this study are listed in the supplement. Methods and figure legends clearly specify samples used in different analyses. Some analyses, as specified, do not include the outlier samples NonDel_MS_MAM, NonDel_FR_47, NonDel_PT_151, NonDel_PA_317, and NonDel_PA_85. Sequence reads for these samples are currently available in public databases (e.g., the European Nucleotide Archives)
Reproducibility	All attempts to repeat experiments were successful.

Randomization	This case study does not involve experimental groups or require randomized treatment or measurement.
Blinding	Operator bias is not relevant to the population genetic analyses performed in this study.

Did the study involve field work? ☐ Yes ☒ No

Reporting for specific materials, systems and methods

We require information from authors about some types of materials, experimental systems and methods used in many studies. Here, indicate whether each material, system or method listed is relevant to your study. If you are not sure if a list item applies to your research, read the appropriate section before selecting a response.

Materials & experimental systems

n/a	Involved in the study	
☒	☐	Antibodies
☒	☐	Eukaryotic cell lines
☒	☐	Palaeontology and archaeology
☐	☒	Animals and other organisms
☒	☐	Human research participants
☒	☐	Clinical data
☒	☐	Dual use research of concern

Methods

n/a	Involved in the study	
☒	☐	ChIP-seq
☒	☐	Flow cytometry
☒	☐	MRI-based neuroimaging

Animals and other organisms

Policy information about studies involving animals; ARRIVE guidelines recommended for reporting animal research

Laboratory animals	The study did not involve laboratory animals.
Wild animals	The study did not involve wild animals.
Field-collected samples	The study did not involve samples collected in the field.
Ethics oversight	The Leishmania strains analysed in this consolidated genomic analysis were derived from the Leishmania Collection from Oswaldo Cruz Foundation (CLIOC), an international cryobank. In all cases Leishmania were isolated from patients as part of normal diagnosis and treatment with no unnecessary invasive procedures and with written and/or verbal consent recorded at the time of clinical examination. All samples were used for research purposes only and the data were analyzed anonymously. Only previously gathered samples from humans and dogs have been used in this study. The activities performed with the Leishmania strains were duly registered in SisGen (Sistema Nacional de Gestao do Patrimonio Genetico) under number A5C0A32, as determined by article 20 of Decree No. 8.772, in accordance with the Brazilian Biodiversity Law (Law No. 13.123/ 2015).

Note that full information on the approval of the study protocol must also be provided in the manuscript.
